# Exploration of haplotype research consortium imputation for genome-wide association studies in 20,032 Generation Scotland participants

**DOI:** 10.1186/s13073-017-0414-4

**Published:** 2017-03-07

**Authors:** Reka Nagy, Thibaud S. Boutin, Jonathan Marten, Jennifer E. Huffman, Shona M. Kerr, Archie Campbell, Louise Evenden, Jude Gibson, Carmen Amador, David M. Howard, Pau Navarro, Andrew Morris, Ian J. Deary, Lynne J. Hocking, Sandosh Padmanabhan, Blair H. Smith, Peter Joshi, James F. Wilson, Nicholas D. Hastie, Alan F. Wright, Andrew M. McIntosh, David J. Porteous, Chris S. Haley, Veronique Vitart, Caroline Hayward

**Affiliations:** 1 MRC Human Genetics Unit, University of Edinburgh, Institute of Genetics and Molecular Medicine, Western General Hospital, Crewe Road, Edinburgh, EH4 2XU UK; 2 Centre for Genomic and Experimental Medicine, University of Edinburgh, Institute of Genetics and Molecular Medicine, Western General Hospital, Edinburgh, UK; 3 Edinburgh Clinical Research Facility, University of Edinburgh, Edinburgh, UK; 4 Division of Psychiatry, University of Edinburgh, Royal Edinburgh Hospital, Edinburgh, UK; 5 Farr Institute of Health Informatics Research, Edinburgh, UK; 6 Centre for Cognitive Ageing and Cognitive Epidemiology, Department of Psychology, University of Edinburgh, Edinburgh, UK; 7 Division of Applied Health Sciences, University of Aberdeen, Aberdeen, UK; 8 Division of Cardiovascular and Medical Sciences, University of Glasgow, Glasgow, UK; 9 Medical Research Institute, University of Dundee, Dundee, UK; 10 Usher Institute of Population Health Sciences and Informatics, University of Edinburgh, Edinburgh, EH8 9AG UK

**Keywords:** Genome-wide association studies (GWAS), Electronic health records, Imputation, Quantitative trait, Genetics, Urate, Heart rate, Glucose, Haplotype Research Consortium (HRC)

## Abstract

**Background:**

The Generation Scotland: Scottish Family Health Study (GS:SFHS) is a family-based population cohort with DNA, biological samples, socio-demographic, psychological and clinical data from approximately 24,000 adult volunteers across Scotland. Although data collection was cross-sectional, GS:SFHS became a prospective cohort due to of the ability to link to routine Electronic Health Record (EHR) data. Over 20,000 participants were selected for genotyping using a large genome-wide array.

**Methods:**

GS:SFHS was analysed using genome-wide association studies (GWAS) to test the effects of a large spectrum of variants, imputed using the Haplotype Research Consortium (HRC) dataset, on medically relevant traits measured directly or obtained from EHRs. The HRC dataset is the largest available haplotype reference panel for imputation of variants in populations of European ancestry and allows investigation of variants with low minor allele frequencies within the entire GS:SFHS genotyped cohort.

**Results:**

Genome-wide associations were run on 20,032 individuals using both genotyped and HRC imputed data. We present results for a range of well-studied quantitative traits obtained from clinic visits and for serum urate measures obtained from data linkage to EHRs collected by the Scottish National Health Service. Results replicated known associations and additionally reveal novel findings, mainly with rare variants, validating the use of the HRC imputation panel. For example, we identified two new associations with fasting glucose at variants near to Y_RNA and WDR4 and four new associations with heart rate at SNPs within CSMD1 and ASPH, upstream of HTR1F and between PROKR2 and GPCPD1. All were driven by rare variants (minor allele frequencies in the range of 0.08–1%). Proof of principle for use of EHRs was verification of the highly significant association of urate levels with the well-established urate transporter SLC2A9.

**Conclusions:**

GS:SFHS provides genetic data on over 20,000 participants alongside a range of phenotypes as well as linkage to National Health Service laboratory and clinical records. We have shown that the combination of deeper genotype imputation and extended phenotype availability make GS:SFHS an attractive resource to carry out association studies to gain insight into the genetic architecture of complex traits.

**Electronic supplementary material:**

The online version of this article (doi:10.1186/s13073-017-0414-4) contains supplementary material, which is available to authorized users.

## Background

Generation Scotland is a multi-institution collaboration that has created an ethically sound, family-based and population-based resource for identifying the genetic basis of common complex diseases [1–3]. The Scottish Family Health Study component (GS:SFHS) has DNA and sociodemographic, psychological and clinical data from ~24,000 adult volunteers from across Scotland. The ethnicity of the cohort is 99% Caucasian, with 96% born in the UK and 87% in Scotland. Features of GS:SFHS include the family-based recruitment, breadth and depth of phenotype information, ‘broad’ consent from participants to use their data and samples for a wide range of medical research and for re-contact, and consent and mechanisms for linkage of all data to comprehensive routine healthcare records. These features were designed to maximise the power of the resource to identify, replicate or control for genetic factors associated with a wide spectrum of illnesses and risk factors [3].

GS:SFHS can also be utilised as a longitudinal cohort due to the ability to link to routine Scottish National Health Service (NHS) data. Electronic Health Record (EHR) linkage uses the ten-digit community health index (CHI) number, a unique identifying number allocated to every person in Scotland registered with a General Practitioner (GP), and used for all NHS procedures (registrations, attendances, samples, prescribing and investigations). This unique patient identifier allows healthcare records for individuals to be linked across time and location [4]. The population is relatively stable with comparatively low levels of geographic mobility and there is relatively little uptake of private healthcare in the population. Few countries, other than Scotland, have health service information which combines high quality data, consistency, national coverage and the ability to link data to allow for genetic and clinical patient-based analysis and follow-up.

The Haplotype Reference Consortium (HRC) dataset is a large haplotype reference panel for imputation of genetic variants in populations of European ancestry, recently made available to the research community [5]. Within a simulated genome-wide association study (GWAS) dataset, it allowed an increased rate of accurate imputation at minor allele frequencies as low as 0.1%, which will allow better interrogation of genetic variation across the allele spectrum. A selected subset of 428 GS:SFHS participants had their exomes sequenced at high depth and contributed reference haplotypes to the HRC dataset, making it ideal for more accurate imputation of this cohort [6].

This paper describes genome-wide association analysis of over 20,000 GS:SFHS participants using two genetic datasets (common, genotyped Single Nucleotide Polymorphisms ﻿(SNPs) and HRC-imputed data) across a range of medically relevant quantitative phenotypes measured at recruitment in research clinics. To illustrate the quality and potential of the many EHR linkage-derived phenotypes available, we selected serum urate as an exemplar due to its direct association with disease, gout, and its strong well-studied genetic associations. About 10% of people with hyperuricemia develop gout, an inflammatory arthritis that results from deposition of monosodium urate crystals in the joint. Genome-wide meta-analyses have identified 31 genome-wide significant urate-associated SNPs, with *SLC2A9* alone explaining ~3% of the phenotypic variance [7].

## Methods

### Sample selection

Selection criteria for genome-wide genotype analysis of the participants were: Caucasian ethnicity; born in the UK (prioritising those born in Scotland); and full phenotype data available from attendance at a Generation Scotland research clinic. The participants were also selected to have consented for their data to be linkable to their NHS electronic medical records using the CHI number. The GS:SFHS genotyped set consisted of 20,195 participants, before quality control exclusions.

### DNA extraction and genotyping

Blood (or occasionally saliva) samples from GS:SFHS participants were collected, processed and stored using standard operating procedures and managed through a laboratory information management system at the Edinburgh Clinical Research Facility, University of Edinburgh [8]. DNA was quantitated using picogreen and diluted to 50 ng/μL; 4 μL were then used in genotyping. The genotyping of the first 9863 samples used the Illumina HumanOmniExpressExome-8 v1.0 BeadChip and the remainder were genotyped using the Illumina HumanOmniExpressExome-8 v1.2 BeadChip, with Infinium chemistry for both [9].

### Phenotype measures

Measurement of total cholesterol, HDL cholesterol, urea and creatinine was from serum prepared from 5 mL of venous blood collected into a tube containing clot activator and gel separator at the time of the visit by the participant to the research clinic. For glucose measurement, 2 mL of venous blood was collected in a sodium fluoride/potassium oxalate tube, with fasting duration recorded. Resting heart rate (pulse) was recorded using an Omron digital blood pressure monitor. Two readings were taken and the second reading was used in the analyses. All other cardiometabolic and anthropometric phenotype measures (see Table 1) are described in [3].

**Table 1:** Top GWAS hits

Baseline characteristic	*N*	dbSNP ID	Minor allele frequency	*p* value	Gene	Imputation quality	Gene association reported previously?	Region significant in genotyped data?
Cardiometabolic								
Diastolic blood pressure	19,546	rs142892876	0.0010	4.97E-08	*CNTN6*	0.75	No	No
		rs528908640	0.0005	1.93E-08	*OPA1*	0.80	No	No
		rs568998724	0.0007	2.91E-08	*-*	0.78	No	No
		rs187680191	0.0006	2.94E-09	*NRG4*	0.51	No	No
Systolic blood pressure	19,547	None			None			
Pulse pressure	19,546	None			None			
Heart rate	19,920	rs9970334	0.4474	4.38E-08	*ICMT*	0.90	Yes	No
		rs755291044	0.0017	1.80E-08	*-*	0.90	No	No
		rs145669495	0.0022	2.01E-08	*CSMD1*	0.90	No	No
		rs142916219	0.0037	2.21E-08	*ASPH*	0.85	No	No
		rs365990	0.3637	4.04E-10	*MYH6*	0.99	Yes	GWS
		rs148397504	0.0008	3.21E-09	*-*	0.45	No	No
Biochemistry								
Serum creatinine	16,347	rs548873184	0.0010	1.47E-08	*LINC00626*	0.96	No	No
		rs573421908	0.0027	1.35E-08	*SLC35F3*	0.80	Yes	No
		rs62412107	0.0660	1.87E-08	*-*	0.79	No	No
		rs3812036	0.2301	1.13E-10	*SLC34A1*	0.96	Yes	GWS
Fasting plasma glucose (with diabetics)	16,174	rs560887	0.2907	6.02E-68	*G6PC2*	1.00	Yes	GWS
		rs9873618	0.2871	9.83E-12	*SLC2A2*	0.99	Yes	GWS
		rs917793	0.1831	2.51E-24	*YKT6*	0.98	Yes	GWS
		rs13266634	0.3153	3.66E-11	*SLC30A8*	1.00	Yes	GWS
		rs533883198	0.0027	3.86E-08	*-*	0.84	No	No
		rs7981781	0.2337	1.40E-08	*PDX1*	0.98	Yes	GWS
		*rs370189685*	0.0014	7.32E-09	*WDR4*	0.63	No	No
Fasting plasma glucose (diabetics removed)	15,226	rs79687284	0.0364	1.87E-08	*-*	0.78	Yes	GWS
		rs780095	0.4267	8.20E-09	*GCKR*	1.00	Yes	GWS
		rs560887	0.2907	2.09E-75	*G6PC2*	1.00	Yes	GWS
		rs8192675	0.2839	8.41E-11	*SLC2A2*	1.00	Yes	GWS
		rs917793	0.1831	1.46E-28	*YKT6*	0.98	Yes	GWS
		rs11558471	0.3227	4.63E-13	*SLC30A8*	1.00	Yes	GWS
		rs143399767	0.0108	1.42E-08	*Y_RNA*	0.89	No	No
		rs7981781	0.2337	5.01E-10	*PDX1*	0.98	Yes	GWS
		rs370189685	0.0014	2.75E-08	*WDR4*	0.63	No	Suggestive
HDL cholesterol	19,223	rs149963466	0.0016	3.18E-08	*-*	0.76	No	No
		rs76183280	0.0048	4.14E-08	*AC016735.2*	0.78	No	No
		*rs4841132*	0.0925	1.08E-08	*RP11-115 J16.1*	1.00	Yes	Suggestive
		rs15285	0.2675	1.16E-18	*LPL*	1.00	Yes	GWS
		rs2740488	0.2745	2.53E-08	*ABCA1*	1.00	Yes	GWS
		*rs138326449*	0.0032	2.92E-20	*APOC3*	0.85	Yes	No
		rs114529226	0.0038	6.98E-09	*IGHVII-33-1*	0.64	No	No
		rs261290	0.3442	2.78E-25	*ALDH1A2*	1.00	Yes	GWS
		rs3764261	0.3261	1.40E-113	*CETP*	1.00	Yes	GWS
		*rs143264468*	0.0010	1.99E-09	*LRRC29*	0.81	Yes	Suggestive
		rs72836561	0.0294	1.55E-11	*CD300LG*	0.87	Yes	No
		*rs149615216*	0.0119	3.20E-09	*LIPG*	0.97	Yes	Suggestive
		rs116843064	0.0230	5.57E-10	*ANGPTL4*	0.84	Yes	No
		rs7412	0.0779	5.95E-14	*APOE*	0.98	Yes	GWS
		*rs453755*	0.2480	3.22E-08	*LILRA3*	0.92	Yes	No
		*rs435306*	0.2547	2.87E-08	*PLTP*	1.00	Yes	Suggestive
Cholesterol	19,259	rs11591147	0.0169	1.83E-17	*PCSK9*	0.98	Yes	GWS
		rs10889333	0.3601	2.12E-10	*DOCK7*	1.00	Yes	GWS
		rs12740374	0.2288	8.19E-22	*CELSR2*	1.00	Yes	GWS
		rs672889	0.1356	3.77E-16	*-*	1.00	Yes	GWS
		rs75331444	0.0720	1.87E-11	*ABCG8*	0.99	Yes	GWS
		rs12916	0.3970	6.35E-11	*HMGCR*	0.99	Yes	GWS
		rs74617384	0.0838	3.02E-09	*LPA*	0.93	Yes	No
		*rs4841133*	0.0929	2.51E-09	*RP11-115 J16.1*	1.00	Yes	Suggestive
		*rs2000999*	0.1776	7.01E-09	*HPR*	0.99	Yes	Suggestive
		rs10412048	0.1086	5.00E-25	*-*	0.98	Yes	GWS
		rs7412	0.0779	5.22E-94	*APOE*	0.98	Yes	GWS
Urea	19,293	*rs760077*	0.4247	6.24E-09	*MTX1*	0.98	Yes	Suggestive
		rs16862780	0.1574	3.03E-10	*RP11-132 N15.3*	1.00	Yes	GWS
		*rs112647987*	0.0680	3.07E-08	*-*	0.99	No	No
		rs6950388	0.1872	1.57E-08	*UNCX*	0.95	Yes	GWS
		*rs10224210*	0.2799	5.71E-09	*PRKAG2*	0.92	Yes	Suggestive
Anthropometric								
Body mass index	19900	rs73139123	0.1830	1.34E-09	*-*	0.96	Yes	GWS
		rs10498218	0.0012	3.98E-08	*COL4A4*	0.84	Yes	No
		rs149913955	0.0059	2.18E-08	*RP11-624 L4.1*	0.74	No	No
		rs571835655	0.0011	6.61E-09	*-*	0.82	No	No
		rs55872725	0.3951	5.71E-21	*FTO*	1.00	Yes	GWS
Height	19,965	rs146949893	0.0031	4.49E-08	*RP1-35C21.2*	0.72	No	No
		rs558671668	0.0062	2.53E-08	*RP11-317P15.6*	0.80	No	No
		rs6765866	0.0007	2.05E-08	*CMTM8*	0.59	No	No
		rs1991431	0.4338	5.25E-13	*ZBTB38*	1.00	Yes	GWS
		*rs35362908*	0.1006	7.04E-09	*LCORL*	0.73	Yes	No
		rs552283803	0.0016	3.99E-08	*ARHGAP24*	0.79	No	No
		rs755546258	0.0007	2.58E-08	*DAP*	0.72	No	No
		*rs72742734*	0.0537	3.61E-08	*NPR3*	1.00	Yes	Suggestive
		rs554379257	0.0006	4.83E-08	*CTD-2023 N9.1*	0.44	No	No
		rs7766641	0.2551	3.32E-13	*HIST1H2BE*	1.00	Yes	GWS
		rs57026767	0.1550	4.50E-11	*C6orf1*	1.00	Yes	GWS
		rs566773279	0.0005	1.17E-08	*-*	0.69	No	No
		rs1490384	0.4851	7.09E-10	*-*	1.00	Yes	GWS
		rs7753012	0.3072	7.60E-14	*GPR126*	0.99	Yes	GWS
		rs184469050	0.0088	1.58E-08	*-*	0.89	No	No
		rs144225905	0.0010	2.13E-09	*-*	0.46	No	No
		rs7952436	0.0896	1.91E-12	*KDM2A*	0.88	Yes	No
		rs634552	0.1365	2.30E-08	*SERPINH1*	0.98	Yes	GWS
		rs76895963	0.0285	3.43E-08	*CCND2*	0.78	Yes [48, 49]	No
		rs770307181	0.0005	1.09E-08	*-*	0.50	No	No
		rs139770682	0.0005	4.55E-08	*-*	0.72	No	No
		rs11614062	0.1943	2.09E-08	*SOCS2-AS1*	0.99	Yes	GWS
		rs75061684	0.0006	5.33E-10	*-*	0.49	No	No
		*rs16942323*	0.0344	1.09E-11	*ACAN*	0.93	Yes	No
		rs8096254	0.2598	4.32E-12	*CABLES1*	1.00	Yes	GWS
		rs6060402	0.3585	2.80E-13	*-*	0.98	Yes	GWS
Waist-to-hip ratio	19,695	*rs72959041*	0.0566	2.54E-14	*RSPO3*	0.90	Yes	Suggestive
		rs149924309	0.0023	3.70E-08	*-*	0.81	No	No
		rs187209742	0.0023	4.91E-08	*SERPINA10*	0.70	No	No
		rs751156121	0.0006	1.29E-08	*-*	0.78	No	No
Body fat	19,480	rs10921288	0.0235	1.04E-08	*-*	0.99	No	GWS
		rs142101835	0.0022	3.25E-08	*IRS1*	0.69	Yes	No
		rs560546550	0.0007	3.17E-09	*WDR41*	0.89	No	No
		rs571835655	0.0011	2.03E-08	*-*	0.82	No	No
		rs55872725	0.3951	5.55E-16	*FTO*	1.00	Yes	GWS
		rs141793746	0.0030	3.31E-08	*DYM*	0.86	No	No
NHS EHR linkage								
Serum urate	2077	rs6449213	0.1652	1.93E-17	*SLC2A9*	1.00	Yes	GWS
		rs75869162	0.0054	1.57E-08	*FAM134B*	0.80	No	No
		rs141208451	0.0053	3.13E-09	*RP11-430H10.4*	0.86	No	No
		rs187171029	0.0060	1.84E-08	*ZNF160*	0.91	No	No

Summary of the baseline characteristics of the GS:SFHS sub-cohort of 20,032 analysed by GWAS, with genome-wide significant markers from the imputed GWAS listed. We indicate known associations in published research or present in the NHGRI GWAS Catalog within 100 kb of the sentinal SNP reported here. The column called ‘Region significant in genotyped data?’ indicates whether any SNPs within 500 kb of the reported SNP reach genome-wide significance (GWS, *p* < 5*10^–8^) or suggestive significance (Suggestive, *p* < 10^–5^) in the genotyped data

The EHR biochemistry dataset was extracted on 28^th^ September 2015 and covers 11,125 participants. EHR data are held in the Tayside Safe Haven, which is fully accredited and utilises a VMware Horizon client environment. Data are placed on a server within a secure IT environment, where the data user is given secure remote access for its analysis [4]. For serum urate, records were available from October 1988 to August 2015. Any data entries in the EHR relating to pregnancy (keywords one or more of ‘pregna/labour/GEST/PET’, total of 117 entries in the urate dataset), were manually removed, as data obtained during pregnancy are usually not included in a GWAS. Many of the participant IDs have multiple readings, spread over time. For extraction of serum urate data for analysis, the highest reading was used, as a high reading would trigger a treatment (such as allopurinol) to lower the urate level, which is then checked by the clinician requesting a subsequent test.

### Genotype data quality control

Genotyping quality control was performed using the following procedures: individuals with a call rate less than 98% were removed, as were SNPs with a call rate less than 98% or Hardy-Weinberg equilibrium *p* value less than 1 × 10^–6^. Mendelian errors, determined using relationships recorded in the pedigree, were removed by setting the individual-level genotypes at erroneous SNPs to missing. Ancestry outliers who were more than six standard deviations away from the mean, in a principal component analysis of GS:SFHS [10] merged with 1092 individuals from the 1000 Genomes Project [11], were excluded. A total of 20,032 individuals (8227 male participants and 11,805 female participants) passed all quality control thresholds. The number of genotyped autosomal SNPs that passed all quality control parameters was 604,858.

### Pedigree correction

Sample identity was verified by comparing the genetic and recorded gender in the first instance and pedigrees were checked for unknown or incorrectly recorded relationships based on estimated genome-wide identity-by-descent (IBD).

Unrecorded first-degree or second degree relationships (calculated IBD ≥ 25%) were identified and entered into the pedigree. Pedigree links to first-degree or second-degree relatives were broken or adjusted if the difference between the calculated and expected amount of IBD was ≥ 25%. After these corrections, any remaining pedigree outliers as determined by examination of the plots of expected versus observed IBD sharing were identified and corrected in the pedigree. Due to some missing parental genotypes, autosomal SNP sharing was not always enough to unambiguously determine whether individuals were related through the maternal or paternal line. In such cases, mitochondrial and/or Y-chromosome markers were compared to help determine the correct lineage.

The full pedigree contains 42,662 individuals (22,383 female participants) in 6863 families, across five generations (average 2.34 generations per family). Family sizes were in the range of 1–66 individuals, with an average of 6.22 individuals per family. The final genotyped dataset contains 9853 parent–child pairs, 8495 full siblings (52 monozygotic twins), 381 half siblings, 848 grandparent–grandchild pairs, 2443 first cousins and 6599 avuncular (niece/nephew–aunt/uncle) relationships.

### Imputation

In order to increase the density of variants throughout the genome, the genotyped data were imputed utilising the Sanger Imputation Service [12] using the HRC panel v1.1 [5, 13]. This exome sequence data will have greatly improved imputation quality across the whole cohort. Autosomal haplotypes were checked to ensure consistency with the reference panel (strand orientation, reference allele, position) then pre-phased using Shapeit2 v2r837 [14, 15] using the Shapeit2 duohmm option11 [16], taking advantage of the cohort family structure in order to improve the imputation quality [17]. Monogenic and low imputation quality (INFO < 0.4) variants were removed from the imputed dataset leaving 24,111,857 variants available for downstream analysis.

### Phenotype quality control and exclusions

Prior to analysis, extreme outliers (those with values more than three times the interquartile distances away from either the 75th or the 25th percentile values) were removed for each phenotypic measure to account for errors in quantification and to remove individuals not representative of normal variation within the population. Approximately 4000 glucose measures were from people who had not fasted for at least 4 h, so these were excluded from the fasting glucose analysis. Additionally, 948 individuals were identified as having diabetes, as determined from self-reporting at the time of sample collection or from EHR-extracted diagnosis of diabetes at any time. Apparent non-diabetics with glucose measures > 7 mmol/L were also removed. Analysis of glucose was performed on both the full fasting dataset and the same dataset excluding diabetics and high glucose outliers.

### Heritability

Heritabilities were estimated for the same phenotype values that were used to run the GWAS. The ‘polygenic’ command in SOLAR version 8.1.1 [18] was used to estimate heritability based on the social pedigrees (no genetic information was used here). The ‘polygenic’ command in the GenABEL R package [19] was used to calculate genetic kinship-based heritability. The standard errors for this latter heritability estimate were obtained by re-running the ‘polygenic’ command and fixing the heritability to 0. The difference between the two estimates yields a one-sided test with a Chi-square distribution with one degree of freedom.

### Genome-wide associations

Genome-wide associations were performed on both genotyped and imputed data. For the HRC-imputed data, only results from variants with a minor allele count of 20 in our sample (or minor allele frequency [MAF] of 0.05%) were considered. For the common variant genotyped data, no MAF cutoff was used. For each phenotype, an additive model for the fitted SNP fixed effect was set up incorporating the same covariates as described in the relevant published meta-analyses or by direct assessment where no prior meta-analysis analysis plan was available (full details in Additional file 1: Table S1) and a random polygenic effect accounting for relatedness among participants. Some phenotypes (as indicated in Additional file 1: Table S1) were inverse-normal transformed to ensure normal distribution of the model’s residuals, using the ‘rntransform’ function in the GenABEL R package [19]. Different GWAS analysis programs were used for the genotype and imputed data to utilise available computational resources most efficiently, but both pipelines account for relatedness.

For the genotype data, the ‘mmscore’ function of GenABEL was used for the genome-wide association test under an additive model. This score test for family-based association takes into account relationship structure and allows unbiased estimations of SNP allelic effect when relatedness is present between individuals. The relationship matrix used in this analysis was generated by the ‘ibs’ function of GenABEL (using weight = ‘freq’ option), which uses genomic data to estimate the realized pair-wise kinship coefficients.

Due to their larger size, the sets of associations with the HRC imputed variants were performed with the software RegScan v0.2 [20]. The pgresidualY estimated from the polygenic function in GenABEL was used for association analysis. The effect size, standard errors and *p* values were thereafter corrected to account for relatedness using the GRAMMAR-Gamma factors also provided by the ‘polygenic’ function [21]. The significance threshold for the genotype and imputed data was set at *p* < 5 × 10^–8^.

## Results

### Heritability

Genetic and social pedigree-based heritabilities were estimated for the phenotypes detailed in Table 1 and are shown in Additional file 2: Figure S1 and Additional file 1: Table S2, along with heritabilities previously described for the same traits (where available) in the literature. The heritabilities of our phenotypes are generally in alignment with those quoted in the literature, except for pulse pressure, whose heritability in our data (0.13, SE 0.01) is approximately half of the heritability quoted in the literature (0.24, SE 0.08) [22]. Conversely, our estimates of the heritability of serum creatinine (0.44, SE 0.01) are more than twice the heritability quoted in the literature (0.19, SE 0.07) [23].

### Genome-wide association studies

We selected four cardiometabolic, six biochemical and four anthropometric quantitative traits to evaluate GWAS outputs from: (1) directly genotyped and (2) HRC-imputed data. The chosen traits are diastolic blood pressure, systolic blood pressure, pulse pressure, heart rate, serum creatinine, fasting plasma glucose, HDL cholesterol, total cholesterol, urea, urate, body mass index, height, waist-to-hip ratio and body fat percentage. The majority of these traits have strong genetic associations when analysed within large multi-cohort meta-analyses, therefore, any genome-wide associations detected in the GS:SFHS cohort can be compared with the established body of knowledge.

Sentinel variants for all the independent genome-wide significant association signals for each phenotype are listed in Table 1, together with their imputation quality if they were not directly genotyped and whether an association signal had previously been reported within ±500 kb. All significant findings were checked against the National Human Genome Research Institute catalogue of published GWAS [24] and, if not present there, were searched for in published papers and other online resources. All SNPs showing trait associations exceeding the threshold for genome-wide significance are reported in Additional files 3, 4, 5, 6, 7, 8, 9, 10, 11, 12, 13, and 14 and in Miami plots, with results using the directly genotyped and HRC-imputed data opposing each other to reflect the gain brought by imputation (Additional file 2: Figures S2–S14). The Q-Q plots for all analyses are shown in Additional file 2: Figure S15 with inflation factors reported in Additional file 1: Table S3. No phenotype showed significant inflation, indicating that correction for stratification has been adequately applied. Multiple, previously identified, significant findings were obtained for all of the traits except for blood pressure measures (Table 1), validating the quality of both the genotypic and the phenotypic data in GS:SFHS. We identified 37 new independent associations across 12 of the 14 selected research clinic-measured phenotypes including four for diastolic blood pressure. Only four of the sentinel SNPs for the novel signals had a MAF greater than 1% (range: 1.08–6.8%); all others are rare, including 13 very rare with MAF < 10^–3^. All but one (rs10921288, MAF = 0.0235, associated with body fat %) were not directly genotyped. In contrast, of the sentinel SNPs in already reported associated regions, only five had a MAF lower than 1%. These include a previously reported replicated association with the same rare variant, the APOC3 splice variant rs138326449 associated with HDL cholesterol [25].

Taking advantage of the availability of pedigrees for GS:SFHS, we looked at whether some of the rare imputed variants are distributed randomly in the population or whether they segregate within families and related individuals. These results are presented in Additional file 1: Table S4 and support a clustering of these variants in families.

**Fig. 1:**
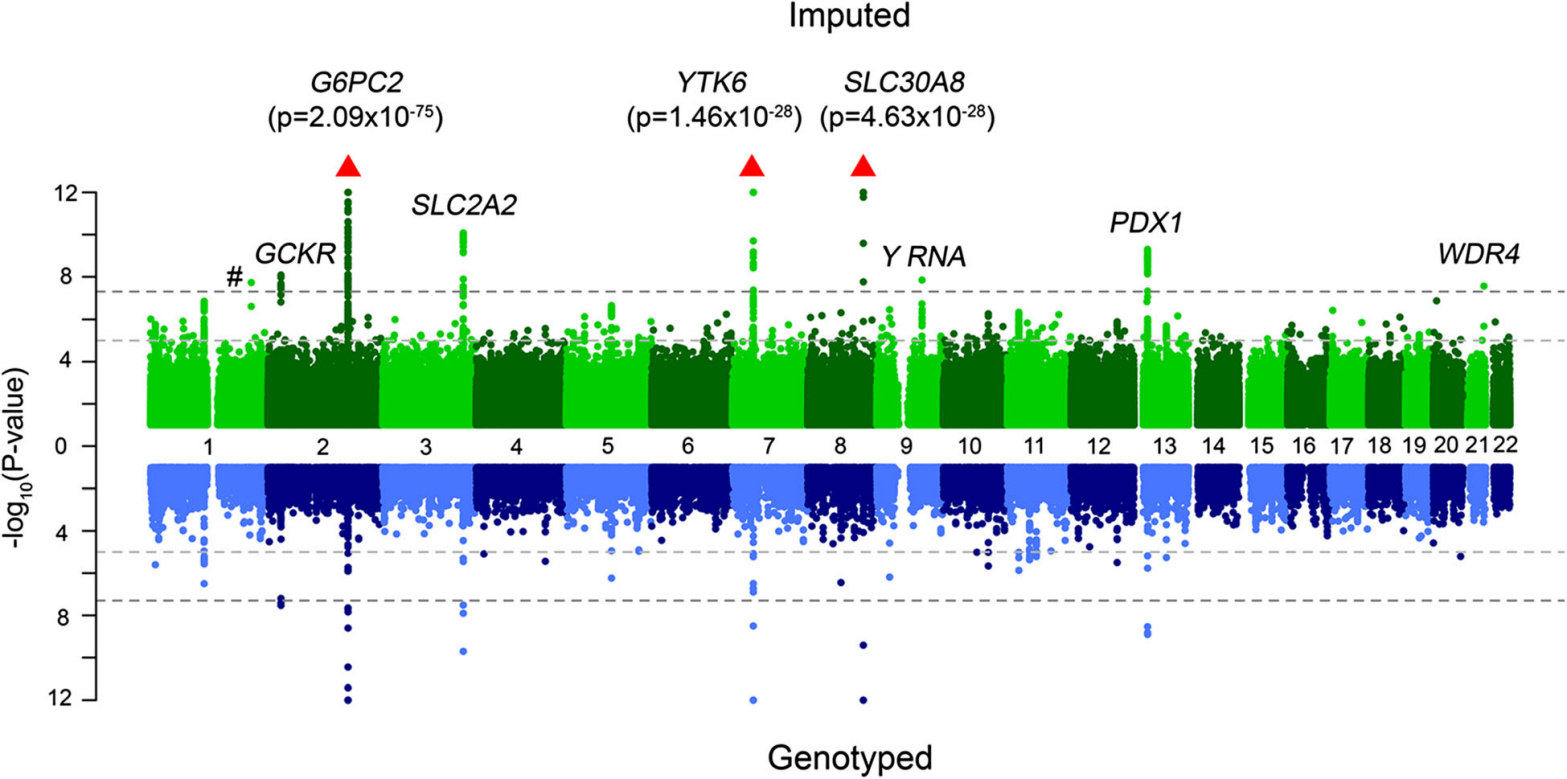
*Miami plot* of fasting plasma glucose. The *top panel* shows the GWAS results using all SNPs imputed to the HRC reference panel, while the *bottom panel* shows only directly genotyped SNPs. In the Miami plot − log10 (*p* value) is plotted on the *y-axis* and chromosomal location is plotted on the *x-axis*. The genome-wide significance threshold after correction for multiple testing (*p* value < 5 × 10^–8^) is indicated by a *dark grey dashed line*, while suggestive significance (*p* value < 10^–5^) is indicated by a *light grey dashed line*. The # symbol denotes a hit in an intergenic region. *Red arrows* indicate SNPs that are not plotted because they have a high –log10 (*p* value) (indicated in brackets)

**Fig. 2:**
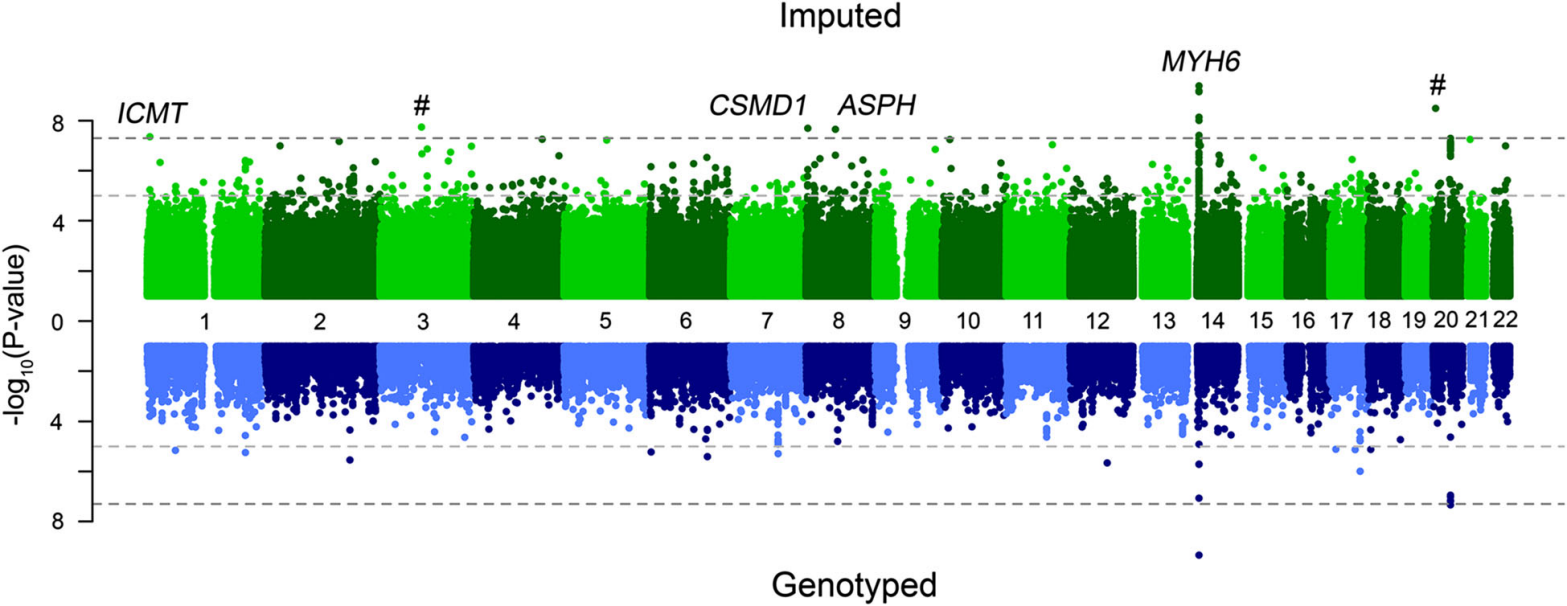
*Miami plot* of resting heart rate. The *top panel* shows the GWAS results using all SNPs imputed to the HRC reference panel, while the *bottom panel* shows only directly genotyped SNPs. In the Miami plot − log10 (*p* value) is plotted on the *y-axis* and chromosomal location is plotted on the *x-axis*. The genome-wide significance threshold after correction for multiple testing (*p* value < 5 × 10^–8^) is indicated by a *dark grey dashed line*, while suggestive significance (*p* value < 10^–5^) is indicated by a *light grey dashed line*. The # symbol denotes a hit in an intergenic region

In Figs. 1 and 2, the results of the GWAS with fasting plasma glucose and resting heart rate, respectively, are depicted in more detail using Miami plots. We identified two novel associations in fasting glucose (Table 2), rs143399767, 2.7 kb upstream of *Y_RNA*, a non-coding RNA which mainly associates with RNA-binding proteins like Ro-60 and insulin-like growth factor 2 messenger RNA binding protein 1 (IGF2BP1) in cytoplasmic ribonucleoprotein complexes [26] and rs370189685 is within an intronic variant of WD repeat domain 4 (*WDR4*), a gene which codes for a transfer RNA-modifying enzyme Both of these are rare variants (minor allele frequencies of 1.08% and 0.1%, respectively). We also replicated known associations in *GCKR*, *G6PC2*, *SLC2A2*, *YKT6*, *SLC30A8* and *PDX1*. We identified four new associations with heart rate (Table 3): rs145669495, a *CSMD1* intronic variant; rs142916219, a *ASPH* intronic variant; and two associations with rs755291044 and rs148397504 in intergenic regions. We additionally replicated known associations at *ICMT* and *MYH6*. The estimated effects of the associated variants in GS:SFHS are shown in Tables 2 and 3 and are compared to those of top hits reported in the meta-analysis summary files (glucose) [27] or the GWAS catalogue (heart rate), respectively. The SNP MAFs from GS:SFHS are also compared against those in the HRC imputation panel.

**Table 2:** Fasting glucose top hits

Gene	SNP	Chr	Position	Effect allele	GS minor allele frequency	HRC MAF	GS *p* value	GS effect size	Meta top SNP	Meta *p* value	GS and meta SNP R^2^	GS and meta SNP D’
*PROX1-AS1*	rs79687284	1	214150821	C	0.036	0.0306	1.87E-08	0.20	rs340874	6.80E-08	0.02	0.99
***GCKR***	**rs780095**	**2**	**27741105**	**G**	**0.427**	**0.4516**	**8.20E-09**	**0.07**	**rs1260326**	**1.26E-24**	**0.72**	**0.93**
***G6PC2***	rs560887*	**2**	**169763148**	**C**	**0.291**	**0.2861**	**2.09E-75**	**0.24**	**rs560887**	**4.68E-100**	**1**	**1**
***SLC2A2***	**rs8192675**	**3**	**170724883**	**C**	**0.284**	**0.3067**	**8.41E-11**	**–0.09**	**rs11920090**	**1.90E-11**	**0.32**	**0.99**
***YKT6***	**rs917793**	**7**	**44245853**	**T**	**0.183**	**0.1766**	**1.46E-28**	**0.17**	**rs4607517**	**1.39E-51**	**1**	**1**
***SLC30A8***	rs11558471*	**8**	**118185733**	**G**	**0.323**	**0.3129**	**4.63E-13**	**–0.09**	**rs11558471**	**3.96E-21**	**1**	**1**
*Y_RNA*	rs143399767	9	96182703	C	0.011	0.0160	1.42E-08	0.36	NA	NA	NA	NA
***PDX1***	**rs7981781**	**13**	**28499962**	**A**	**0.234**	**0.2296**	**5.01E-10**	**0.09**	**rs2293941**	**2.93E-08**	**0.99**	**0.99**
*WDR4*	rs370189685	21	44276432	C	0.001	0.0009	2.75E-08	–1.15	NA	NA	NA	NA

Summary of top hits of the imputed GWAS analysis of fasting plasma glucose (15,226 people after those with diabetes were removed) in Generation Scotland, compared with top hits in a meta-analysis reported in [50]. Starred (*) SNPs indicate the same SNP in the GS and meta-analysis datasets. Entries in bold are within 500,000 bases of a SNP that reached genome-wide significance in the genotyped GWAS analysis. Entries with missing meta-analysis top SNPs (indicated by NA) are novel associations that did not reach significance in the meta-analysis

**Table 3:** Heart rate top hits

Gene	SNP	Chr	Position	Effect allele	GS minor allele frequency	HRC MAF	GS *p* value	GS effect size	GWAS catalog top SNP	GWAS catalog *p* value	GS and meta SNP R^2^	GS and meta SNP D’
*ICMT*	rs9970334	1	6296238	G	0.447	0.4502	4.38E-08	0.70	rs846111	7.00E-40 [51]	0.47	0.98
*-*	rs755291044	3	87751558	A	0.002	-	1.80E-08	8.60	NA	NA	NA	NA
*CSMD1*	rs145669495	8	4102424	G	0.002	0.0024	2.01E-08	7.66	NA	NA	NA	NA
*ASPH*	rs142916219	8	62481520	G	0.004	0.0022	2.21E-08	5.97	NA	NA	NA	NA
***MYH6***	rs365990*	**14**	**23861811**	**G**	**0.364**	**0.3644**	**4.04E-10**	**0.78**	**rs365990**	**5.00E-45** [52]	**1**	**1**
*-*	rs148397504	20	5376623	A	0.001	0.0006	3.21E-09	18.54	NA	NA	NA	NA

Summary of top hits of the imputed GWAS analysis of heart rate in 19,920 Generation Scotland participants, compared with associations reported in the GWAS catalogue. The starred (*) SNP indicates the same SNP in the GS and GWAS catalogue. Entries in bold are within 500,000 bases of a SNP that reached genome-wide significance in the genotyped GWAS analysis. Entries with missing GWAS catalogue top SNPs (indicated by NAs) are novel associations

### GWAS of serum urate extracted from electronic health records

In the 11,125 individuals with NHS EHR biochemistry available, there are 2356 GS:SFHS participants with serum urate measured at least once and a total of 6268 tests. The proportion of participants who have had at least one test recorded for urate is 21%. Of these participants, 214 have been identified as having taken allopurinol, a urate-lowering medication, either through self-reporting at GS:SFHS clinic visit or through NHS prescription data linkage. The highest urate measure from all individuals was used for GWAS.

**Fig. 3:**
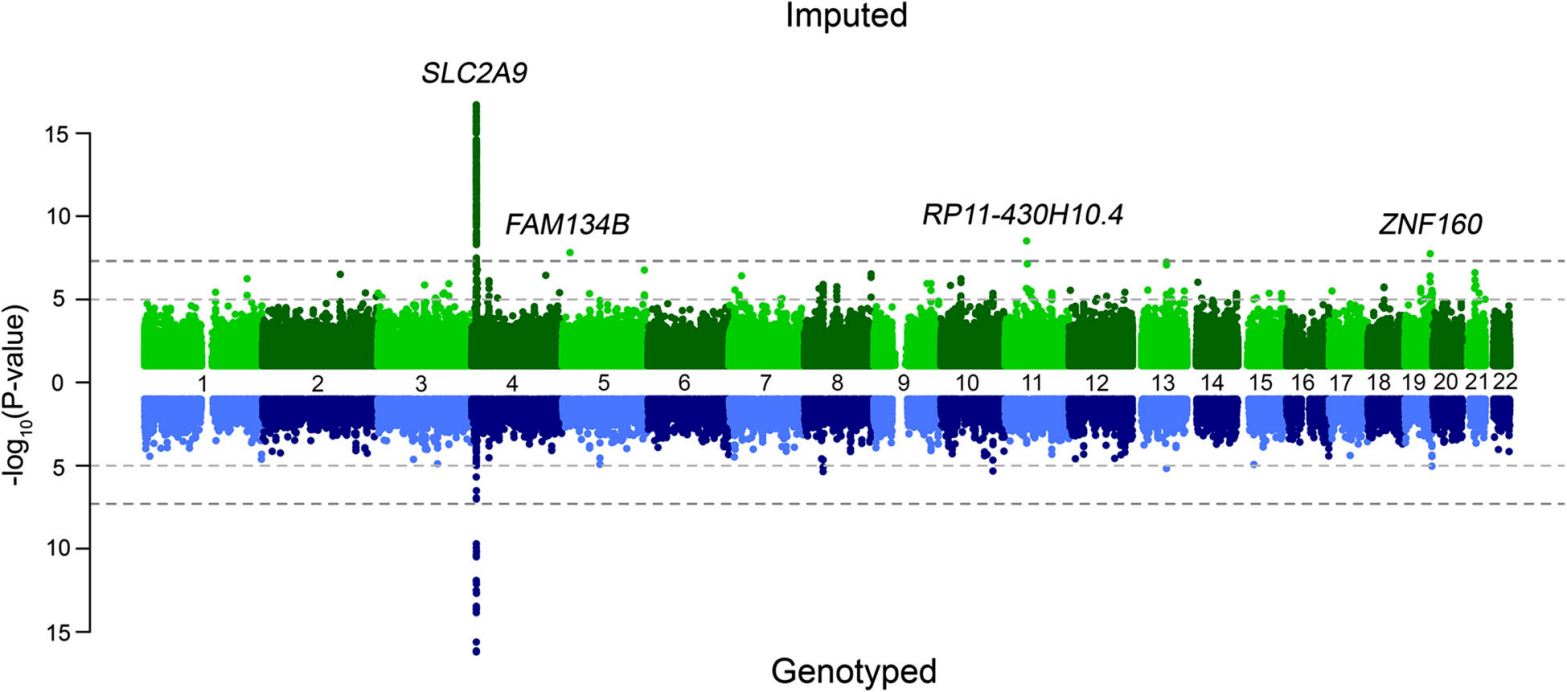
*Miami plot* of uric acid. The *top panel* shows the GWAS results using all SNPs imputed to the HRC reference panel, while the *bottom panel* shows only directly genotyped SNPs. In the Miami plot − log10 (*p* value) is plotted on the *y-axis* and chromosomal location is plotted on the *x-axis*. The genome-wide significance threshold after correction for multiple testing (*p* value < 5 × 10^–8^) is indicated by a *dark grey dashed line*, while suggestive significance (*p* value < 10^–5^) is indicated by a *light grey dashed line*

The GWAS for urate was performed using both genotype and imputed data, taking into account the sex of the participant and adjusting for participant age at the time of the test. The results of these analyses are displayed in Fig. 3. In both analyses, the association with the lowest *p* value was at the well-established *SLC2A9* locus and the most significant SNP was rs6449213 with a *p* value of 7.2 × 10^–17^ in the genotype data and 5.13 × 10^–17^ in the imputed data (Table 4, Fig. 3). This was the only locus reaching genome-wide significance for this trait in the genotyped analysis. Additionally, three loci exceeded our threshold for significance in the imputed analysis – the sentinal SNPs are rs75869162 in RP11-260E18.1-001 (a long non-coding RNA of unknown function), rs141208451 in RP11-958 J22.2 (a novel processed transcript) and RP11-430H10.4 (a long non-coding RNA of unknown function); and rs187171029, an intronic variant in *ZNF160*. All of these new associations are with rare variants (MAF < 1%, range: 0.53–0.6%) not present in the results of the largest serum urate GWAS from the Global Urate Genomics Consortium (GUGC) [28] (Additional file 1: Table S5).

**Table 4:** Uric acid top hits

Gene	SNP	Chr	Position	Effect allele	GS minor allele frequency	HRC MAF	GS *p* value	GS effect size	Meta top SNP	Meta *p* value
***SLC2A9***	**rs6449213**	**4**	**9994215**	**T**	**0.165**	**0.1857**	**1.93E-17**	**0.592**	**rs12498742**	**<1 E − 700**
*FAM134B*	rs75869162	5	16617922	A	0.005	0.0019	1.57E-08	2.24	rs386845	1.18E-02
*RP11-430H10.4*	rs141208451	11	45538920	A	0.005	0.0011	3.13E-09	2.32	rs11038475	7.36E-03
*ZNF160*	rs187171029	19	53599256	T	0.006	0.0040	1.84E-08	2	rs16984293	2.58E-02

Summary of top hits of the imputed GWAS analysis of uric acid in 2077 Generation Scotland participants, compared with top hits in a meta-analysis reported in the GUGC. Top hits were extracted from the region within 100,000 bases of the imputed GWAS top SNP. Entries in bold are within 500,000 bases of a SNP that reached genome-wide significance in the genotyped GWAS analysis

## Discussion

The continued improvement in scale and coverage of haplotype reference panels for use in imputation has opened possibilities for exploration of the contribution of low frequency and rare variants to traits previously analysed in GWAS [25, 29], where the contribution of common variation is better known.

We investigated the use of the recently released HRC imputation set to perform GWAS in a large study of > 20,000 individuals from the GS:SFHS cohort, illustrating both the promise and the challenge of such studies.

### Study advantages

This analysis is performed on the largest single homogeneous population sample, to date. We detected most known genetic associations with common variants (MAF > 5%) using the genotyped dataset alone. For many of these associations, a weaker signal was detected using the genotyped GWAS, which became stronger in imputed SNPs that are presumably more closely linked to the causal variant. We identify such associations in many of the traits reported in Table 1 and the majority are within a known association signal.

The pedigree-based heritability estimates are slightly higher than the heritabilities estimated using the genetic data, which could be because the genotype-based heritability estimation only considers additive genetic effects (but not dominant or epistatic effects). Additionally, the pedigree-based heritability might be capturing the effects of a shared environment between family members living in the same household, which can inflate the heritability estimates.

Imputated data generated most of the association signals with low frequency and rare variants. We investigated, in greater detail, the biological relevance of the associations detected in heart rate.

We identified four new associations with heart rate (Table 3). rs755291044 is located 300 kb upstream of the nearest gene, 5-Hydroxytryptamine Receptor 1 F (*HTR1F*), which codes for a subunit of the serotonin receptor. Serotonin (5-hydroxytryptamine) is known to modulate heart rate and blood pressure through direct vascular effects and indirectly through the sympathetic nervous system [30].

Intronic variant rs145669495 is within the CUB and Sushi Multiple Domains 1 gene (*CSMD1*), which has a reported association with blood pressure in a Korean cohort [31]. While there is only a weak epidemiological correlation between heart rate and blood pressure, it is interesting that we find two genetic loci that affect both phenotypes.

rs142916219 lies within an intron of the Aspartate Beta-Hydroxylate/Junctin gene (*ASPH*), which is a regulator of calcium homeostasis. Some isoforms encoded by this gene localize to the sarcoplasmic reticulum, which is the smooth endoplasmic reticulum found in muscle tissue (including heart muscle). The relationship between calcium concentration and (heart) muscle contraction is well documented [32] and reductions in the level of ASPH have been linked to heart failure and arrhythmia [33].

rs148397504 is 80 kb upstream of the Prokineticin Receptor 2 gene (*PROKR2*) and 150 kb downstream of the glycerophosphocholine Phosphodiesterase 1 gene (*GPCPD1*), within a CTCF binding site. *PROKR2* encodes a receptor for prokineticin, a secreted protein that promotes angiogenesis [34] and heart development [35]. Activation of this receptor leads to calcium mobilization and *PROKR1*, a paralog with unusually high sequence similarity to this receptor, has been associated with insulin-mediated Akt signalling and myocardial fibrosis, diastolic dysfunction and impaired capillary formation [36, 37].

*GPCPD1* (formerly *GDE5*), the upstream gene, is highly expressed in the fetal heart and is involved in skeletal muscle differentiation [38]. We note that this variant has a low imputation quality (0.44), so this association should be treated with caution until it is replicated in another study or is confirmed through direct sequencing in carriers.

It is encouraging that these novel associations lie within, or near, genes that are known, or suspected, to affect cardiac muscle function and morphology, blood pressure and heart rate.

We note that an association which reached genome-wide significance in the genotype data (rs6127466, *p* = 4.58 × 10^–8^) drops to just below the threshold in the HRC-imputed data (*p* = 7.27 × 10^–8^). This SNP lies within the *KIAA1755* gene, which has been found to associate with heart rate in the GWAS catalogue. During quality control, five individuals had their genotypes set to missing at this SNP. These individuals’ genotypes were then imputed, allowing them to be included in the analysis and subsequently altering the *p* value of the association.

We also show here the validity of phenotypes derived from electronic health records in GS:SFHS. The value of EHRs in genomics research is becoming widely recognised (e.g. [39, 40]). The focus to date has largely been on genetic associations with International Classification of Disease (ICD-9 or ICD-10) codes which are available in most EHR systems but successful GWAS of several liver biochemistry measures in 3294 samples from the eMERGE network have recently been described [41].

The anticipated GWAS hits in *SLC2A9* were found for serum urate in this project and validate this EHR data resource as a valuable method of acquiring additional phenotypes for the GS:SFHS cohort. We did not detect significant effects from other known urate loci, such as *ABCG2*, but this is not entirely surprising given that our sample size is much smaller than most consortium meta-analyses. However, the majority show comparable effect size and direction (Additional file 2: Figure S16).

We did, however, detect three new loci with signals driven by rare variants (Table 4).

As well as linking to routine biochemistry, linkage can be made to hospital inpatient episode data (Scottish Morbidity Record, SMR01; ICD-10 codes) and to prescribing data, providing multiple opportunities to further exploit this approach.

## Limitations

### Validation of rare variant imputation

Most of the low frequency and rare variants were imputed and absent from the genotyping arrays and would need to be validated by direct genotyping. We found that the associated rare variants tended to cluster within related individuals, as up to 90% (and on average, 55%) of the carriers of each rare variant reported for heart rate, fasting glucose and serum urate shares a kinship coefficient of greater than 0.05 (are fourth-degree relatives) with at least one other carrier. In fact, most of these pairs of carriers have a kinship coefficient ≥ 0.25 (second-degree relatives) and are assigned to the same family in the pedigree file. This gives some support to the validity of these variants and illustrates the advantage gained by imputing into family-based cohorts. The splice variant rs138326449 (MAF 0.032% in GS:SFHS) in the *APOC3* gene has been validated in a pioneering GWAS UK10K study that first report its association with HDL cholesterol, plasma triglycerides and VLDL levels in the ALSPAC and TwinsUK cohorts [25]. Four other rare variants—rs142101835 (*IRS1*), associated with body fat; rs143264468 (*LRRC29*), associated with HDL cholesterol; rs10498218 (*COL4A4*), associated with BMI; and rs573421908 (*SLC35F3*), associated with serum creatinine—also replicated in our study. In addition, two of the novel loci associated with diastolic blood pressure in our study have been implicated in clinical studies. Polymorphisms in mitochondrial dynamin like GTPase (*OPA1*) were reported to have an age-dependent association with blood pressure and hypertension in a Korean population [42]. Low levels of serum Neuregulin 4 (*NRG4*) were recently shown to be strongly associated with elevated blood pressure and fasting glucose in a Chinese study of over 1200 obese adults [43].

### Validation of novel association

We used a threshold for genome-wide significance of 5 × 10^–8^, as a more stringent one based on the number of independent variants tested and number of traits tested would leave very few of the previously described association signals, listed in Table 1, reaching genome-wide significance while most of those (admittedly those driven by the common variants) have been well replicated in large studies. It is clear that a proportion of the results reported here will be false positives and all novel associations will need replication.

For a more global assessment of our data quality we compared the alleles reported in GS:SFHS HRC imputed data versus the available high quality exome chip data for the same samples (52,007 overlapping SNPs with [maf > = 5e-4]) and found high levels of agreement and a concordance of 95.3% for all SNPs, 98.4% concordance for SNP with a MAF > = 0.01 (20,012 SNPs) and 89% for rarer SNPs (frequency < 0.01) (31,995 SNPs). We have also checked for concordance for the genome-wide significant hits found in our association studies with the available exome sequence data from 864 individuals in GS:SFHS and identified 20 SNPS, all of which had a concordance of at least 97%. Unfortunately, only two rare variants identified in our GWAS—rs142101835 (*IRS1*) and rs138326449 (*APOC3*)—were among these 20 SNPs.

We also made a further attempt to confirm some additional novel variants by checking HRC-imputed results from two other cohorts (ORCADES and VHS) (http://www.orcades.ed.ac.uk/orcades/VHSS.html), but because of the considerably smaller sample sizes (~2000 in each cohort) no rare variants were sufficiently frequent to establish a replication.

Replication in other populations may be difficult for the rare variants because variants such as these of large effect will be kept at low frequency and not found in other disparate populations by the effects of natural selection, which are likely much more pronounced on large effects than on small. However, the precedent of replication of the APOC3 variant rs138326449 is promising and very large study in the UK (such as UKBiobank) where similar imputations will be performed are soon to be available.

## Conclusions

Here, we present the first detailed description of the entire GS:SFHS GWAS dataset. While a subset of Generation Scotland has already served as a valuable co-discovery and replication cohort for genetic associations for a range of traits (e.g. [44–46]), here we demonstrate the stand-alone value of the full cohort through replication of established genetic associations, as well as through the discovery of several novel associations. Although not presented here, the family-based structure of GS:SFHS allows the shared variation between individuals within families to be disentangled into its genetic and environmental components (e.g. [47]).With the growing emphasis on the use of routine administrative health data, studies such as this project become increasingly important in order to provide information on the accuracy and validity of other findings that are based on EHRs.

This dataset is now available for collaborative studies and meta-analyses that are consistent with the original ‘broad’ consent [2].

## Additional files

Additional file 1: Table S1. Phenotypes and covariates*.* **Table S2.** Phenotype heritability. **Table S3.** Lambdas for all traits, calculated using GenABEL’s estlambda median function. **Table S4.** Top hits from GUGC serum urate meta-analysis. **Table S5.** Relatedness of rare variant carriers. (PDF 598 kb)
Additional file 2: Figure S1. Phenotype heritabilities*.* **Figure S2.** Miami plot for height. **Figure S3.** Miami plot for BMI. **Figure S4.** Miami plot for waist circumference. **Figure S5.** Miami plot for waist-to-hip ratio. **Figure S6.** Miami plot for body fat percentage. **Figure S7.** Miami plot for diastolic blood pressure. **Figure S8.** Miami plot for creatinine. **Figure S9.** Miami plot for urea. **Figure S10.** Miami plot for fasting glucose (all individuals included). **Figure S11.** Miami plot for fasting glucose (excluding diabetics and measurements of >7 mmol/L). **Figure S12.** Miami plot for HDL cholesterol. **Figure S13.** Miami plot for total cholesterol. **Figure S14.** Miami plot for total cholesterol adjusted for statin use. **Figure S15.** QQ plots for all traits analysed. **Figure S16.** Comparison of effect sizes for GUGC top hits in the GS:SFHS EHR analysis. (PDF 2160 kb)
Additional file 3: GS_HRC_imputed_Body_Mass_Index_significant_SNPS. (XLSX 36 kb)
Additional file 4: GS_HRC_imputed_Diastolic_Blood_Pressure_significant_SNPS. (XLSX 8 kb)
Additional file 5: GS_HRC_imputed_Heart_Rate_significant_SNPS. (XLSX 9 kb)
Additional file 6: GS_HRC_imputed_Serum_Creatinine_significant_SNPS. (XLSX 10 kb)
Additional file 7: GS_HRC_imputed_Fasting_Glucose (including_diabetics)_significant_SNPS. (XLSX 33 kb)
Additional file 8: GS_HRC_imputed_Fasting_Glucose (excluding_diabetics)_significant_SNPS. (XLSX 36 kb)
Additional file 9: GS_HRC_imputed_HDL_cholesterol_significant_SNPS. (XLSX 71 kb)
Additional file 10: GS_HRC_imputed_Height_significant_SNPS. (XLSX 64 kb)
Additional file 11: GS_HRC_imputed_Total_cholesterol_significant_SNPS. (XLSX 92 kb)
Additional file 12: GS_HRC_imputed_Urea_significant_SNPS. (XLSX 12 kb)
Additional file 13: GS_HRC_imputed_Waist_Hip_Ratio_significant_SNPS. (XLSX 8 kb)
Additional file 14: GS_HRC_imputed_Body_Fat_significant_SNPS. (XLSX 19 kb)

## Abbreviations

CHI: Community health index
EHR: Electronic health record
GS:SFHS: Generation Scotland: Scottish Family Health Study
GWAS: Genome-wide association study
HRC: Haplotype Research Consortium
IBD: Identity-by-descent
MAF: Minor allele frequency
NHS: National Health Service
SNP: Single Nucleotide Polymorphism
