# Supplementary material for: Exploration of haplotype research consortium imputation for genome-wide association studies in 20,032 Generation Scotland participants
Source: Genome Med. 2017 Mar 7;9:23. doi: 10.1186/s13073-017-0414-4 (PMC5339960; doi:10.1186/s13073-017-0414-4)
Supplement: Additional file 1: Table S1. — Phenotypes and covariates. Table S2. Phenotype heritability. Table S3. Lambdas for all traits, calculated using GenABEL’s estlambda median function. Table S4. Top hits from GUGC serum urate meta-analysis. Table S5. Relatedness of rare variant carriers. (PDF 598 kb) [file 13073_2017_414_MOESM1_ESM.pdf]

Additional file 1: Table S1 - Phenotypes and Covariates

This table summarizes the phenotypes used in the GWAS, along with their covariates and whether any normalization was applied. Rank transforming was done using the rntransform (trait~covariates) function of the GenABEL R package. BMI is body mass index

Trait	Units	N	Mean (SD)	Transformation	Covariates
Height	m	19965	1.681 (0.09)	No transformation	sex+age
Body Mass Index	kg/m^2	19900	26.71 (5.24)	Rank transform	sex+age+age*age
Waist-to-Hip Ratio	NA	19695	0.86 (0.09)	Rank transform	sex+age+age*age+bmi
Body Fat	%	19480	30 (9.63)	No transformation	sex+age
Diastolic Blood Pressure	mmHg	19546	80.79 (11.42)	No transformation	sex+age+age*age+bmi
Systolic Blood Pressure	mmHg	19547	131.7 (19.71)	Rank transform	sex+age+age*age+bmi
Pulse Pressure	mmHg	19546	50.87 (13.54)	Rank transform	sex+age+age*age+bmi
Heart Rate	beats per minute	19920	69.9 (11.46)	No transformation	sex+age+age*age+bmi
Serum Creatinine	mg/dl	16347	0.826 (0.18)	Rank transform	age+sex
Urea	mg/dl	19293	14.38 (4.14)	Rank transform	age+sex
Fasting Plasma Glucose (all)	mmol/l	16174	4.773 (0.92)	Rank transform	sex+age+age*age+bmi
Fasting Plasma Glucose (Diabetics excluded)	mmol/l	15226	4.687 (0.46)	Rank transform	sex+age+age*age+bmi
HDL Cholesterol	mg/dl	19223	56.51 (15.87)	Rank transform	sex+age+age*age
Total Cholesterol	mg/dl	19259	196.9 (41.62)	Rank transform	sex+age+age*age
Serum Urate	mg/dl	2077	5.75 (1.84)	No transformation	sex+age

Additional file 1: Table S2 – Phenotype Heritability

Comparison of Pedigree and Genetic heritability estimates with published sources

Phenotype	Literature_h2	Literature_SE	Pedigree_h2	Pedigree_SE	Genetic_h2	Genetic_SE	Lit_ref
Diastolic BP	0.37	0.09	0.29	0.02	0.26	0.01	https://www.ncbi.nlm.nih.gov/pubmed/17278972
Systolic BP	0.34	0.09	0.25	0.02	0.24	0.01	https://www.ncbi.nlm.nih.gov/pubmed/17278972
Pulse Pressure	0.24	0.08	0.12	0.02	0.13	0.01	https://www.ncbi.nlm.nih.gov/pubmed/17278972
Heart Rate	NA	NA	0.30	0.02	0.25	0.01	
Creatinine	0.19	0.07	0.53	0.02	0.44	0.01	https://www.ncbi.nlm.nih.gov/pmc/articles/PMC4330070/
Glucose	0.33	0.12	0.26	0.02	0.22	0.01	http://journals.plos.org/plosgenetics/article?id=10.1371/journal.pgen.1002637
HDL Cholesterol	0.48	0.11	0.59	0.02	0.51	0.00	http://journals.plos.org/plosgenetics/article?id=10.1371/journal.pgen.1002637
Total Cholesterol	0.37	0.13	0.31	0.02	0.27	0.01	http://biorxiv.org/content/biorxiv/suppl/2016/09/09/074310.DC1/074310-1.pdf
BMI	0.42	0.17	0.56	0.02	0.48	0.01	https://www.ncbi.nlm.nih.gov/pmc/articles/PMC3965855/
Height	0.69	0.14	0.92	0.01	0.82	0.00	https://www.ncbi.nlm.nih.gov/pmc/articles/PMC3965855/
Waist Hip Ratio	0.28	0.12	0.28	0.02	0.24	0.01	http://journals.plos.org/plosgenetics/article?id=10.1371/journal.pgen.1002637
Body Fat	NA	NA	0.53	0.02	0.45	0.01	

Additional file 1: Table S3 - Lambdas for all traits, calculated using GenABEL's estlambda median function

**Trait**	**HRC Imputed**	**Genotyped**
Height	1.15	0.98
BMI	1.09	0.99
Waist Circumference	1.07	1.00
Waist:Hip Ratio	1.04	0.99
Body Fat Percentage	1.08	1.00
Diastolic Blood Pressure	1.04	1.00
Heart Rate	1.05	1.00
Serum creatinine	1.07	0.99
Urea	1.04	0.99
Fasting Glucose (all)	1.03	0.99
Fasting Glucose (Diabetics excluded)	1.03	0.99
HDL Cholesterol	1.08	0.98
Total Cholesterol	1.04	0.99
Total Cholesterol (Statin-adjusted)	1.06	0.99
NHS Uric Acid	0.99	1.00

Additional file 1: Table S4 - Top hits from GUGC serum urate meta analysis in GS:SFHS

Gene	SNP	Chr.	Position	Imputation Quality	MAC	MAF	GS:SFHS p-value	GS:SFHS effect size	GUGC p-value	GUGC effect size
PDZK1	rs1471633	1	145723739	0.985	21296	0.488	1.48E-01	0.078	1.40E-26	0.061
TRIM46	rs11264341	1	155151493	0.999	16683	0.419	1.11E-01	-0.087	1.04E-14	-0.048
GCKR	rs1260326	2	27730940	0.998	24806	0.393	7.59E-01	0.017	1.31E-40	0.077
INHBB	rs17050272	2	121306440	0.981	15795	0.401	3.25E-01	0.053	9.36E-09	0.037
ORC4L	rs2307394	2	148716428	1.000	11581	0.286	3.35E-01	-0.056	7.26E-09	-0.035

SFMBT1	rs6770152	3	53100214	0.995	23071	0.426	9.49E-03	-0.139	2.66E-16	-0.048
SLC2A9	rs12498742	4	9944052	0.999	8818	0.204	5.56E-14	0.486	0.00E+00	0.38
ABCG2	rs2231142	4	89052323	0.997	5117	0.133	6.61E-04	0.267	4.43E-116	0.22
TMEM171	rs17632159	5	72431482	0.958	12580	0.329	4.37E-01	-0.044	2.00E-09	-0.038
RREB1	rs675209	6	7102084	0.985	29446	0.270	3.05E-01	0.060	1.38E-21	0.063
SLC17A1	rs1165151	6	25821616	0.999	22616	0.439	2.19E-01	-0.065	4.52E-60	-0.092
VEGFA	rs729761	6	43804571	0.966	28523	0.289	1.27E-02	-0.147	3.05E-12	-0.046
BAZ1B	rs1178977	7	72857049	1.000	8286	0.201	1.69E-01	0.091	6.68E-12	0.05
PRKAG2	rs10480300	7	151406005	0.976	10900	0.264	5.18E-01	-0.039	9.37E-07	0.032
STC1	rs17786744	8	23777006	0.997	17086	0.437	2.93E-01	-0.056	8.82E-08	-0.031
HNF4G	rs2941484	8	76478768	0.998	17069	0.435	2.67E-01	0.057	3.91E-17	0.049
A1CF	rs10821905	10	52646093	0.991	7192	0.171	7.78E-01	0.020	3.45E-12	0.053
SLC16A9	rs1171614	10	61469538	0.974	30921	0.228	8.83E-02	-0.110	6.48E-23	-0.074
SLC22A11	rs2078267	11	64334114	0.973	22341	0.453	1.64E-01	-0.076	8.73E-36	-0.078
NRXN2	rs478607	11	64478063	0.995	34088	0.140	5.04E-01	-0.050	5.31E-10	-0.048
OVOL1	rs642803	11	65560620	0.999	19138	0.463	1.36E-01	-0.081	4.51E-14	-0.043
INHBC	rs3741414	12	57844049	0.983	9325	0.225	2.55E-02	-0.141	9.79E-22	-0.071
ATXN2	rs653178	12	112007756	0.976	19768	0.480	1.35E-01	0.081	2.45E-10	-0.036
UBE2Q2	rs1394125	15	76158983	0.993	14433	0.360	7.42E-01	0.018	9.78E-11	0.043
IGF1R	rs6598541	15	99271135	0.992	25710	0.368	5.00E-01	0.036	5.20E-13	0.044
NFAT5	rs7193778	16	69563890	0.999	33588	0.169	1.21E-01	-0.109	2.36E-08	-0.047

MAF	rs7188445	16	79734987	0.995	12477	0.321	3.82E-01	-0.050	1.15E-07	-0.032
HLF	rs7224610	17	53364788	0.997	24029	0.410	5.78E-01	0.030	4.74E-11	-0.038
BCAS3	rs2079742	17	59465697	0.918	5320	0.136	1.72E-01	0.109	6.24E-09	0.051
QRICH2	rs164009	17	74283669	1.000	16014	0.402	1.26E-01	0.083	7.06E-07	0.029

Additional file 1: Table S5 - Relatedness of rare variant carriers

This table presents the number of people carrying the rare variants for which we have discovered novel associations with fasting glucose, heart rate and serum urate. "N Carriers" refers to the number of people who carry at least one of the rare alleles of these variants. "N Related" refers to the number of carriers who have a kinship coefficient of at least 0.05 with another carrier, "N Family" refers to the subset of "N Related" who additionally also share a family ID with the carrier they are related to.

rsID	N Carriers	N Related (% of carriers)	N Family (% of carriers)
**Fasting Glucose**			
rs143399767	368	275 (75%)	228 (62%)
rs370189685	28	19 (68%)	18 (64%)
**Heart Rate**			
rs755291044	56	37 (66%)	32 (57%)
rs145669495	78	45 (58%)	38 (49%)
rs142916219	119	89 (75%)	75 (63%)
rs148397504	9	8 (89%)	8 (89%)
**Serum Urate**			
rs75869162	16/121	5 (31%)/ 89 (74%)	2 (13%)/ 79 (65%)
rs141208451	17/152	5 (29%)/ 107 (70%)	5 (29%)/ 91 (60%)
rs187171029	22/337	4 (18%)/	2 (9%)/
