# Supplementary material for: Exploration of haplotype research consortium imputation for genome-wide association studies in 20,032 Generation Scotland participants
Source: Genome Med. 2017 Mar 7;9:23. doi: 10.1186/s13073-017-0414-4 (PMC5339960; doi:10.1186/s13073-017-0414-4)
Supplement: Additional file 2: Figure S1. — Phenotype heritabilities. Figure S2. Miami plot for height. Figure S3. Miami plot for BMI. Figure S4. Miami plot for waist circumference. Figure S5. Miami plot for waist-to-hip ratio. Figure S6. Miami plot for body fat percentage. Figure S7. Miami plot for diastolic blood pressure. Figure S8. Miami plot for creatinine. Figure S9. Miami plot for urea. Figure S10. Miami plot for fasting glucose (all individuals included). Figure S11. Miami plot for fasting glucose (excluding diabetics and measurements of >7 mmol/L). Figure S12. Miami plot for HDL cholesterol. Figure S13. Miami plot for total cholesterol. Figure S14. Miami plot for total cholesterol adjusted for statin use. Figure S15. QQ plots for all traits analysed. Figure S16. Comparison of effect sizes for GUGC top hits in the GS:SFHS EHR analysis. (PDF 2160 kb) [file 13073_2017_414_MOESM2_ESM.pdf]

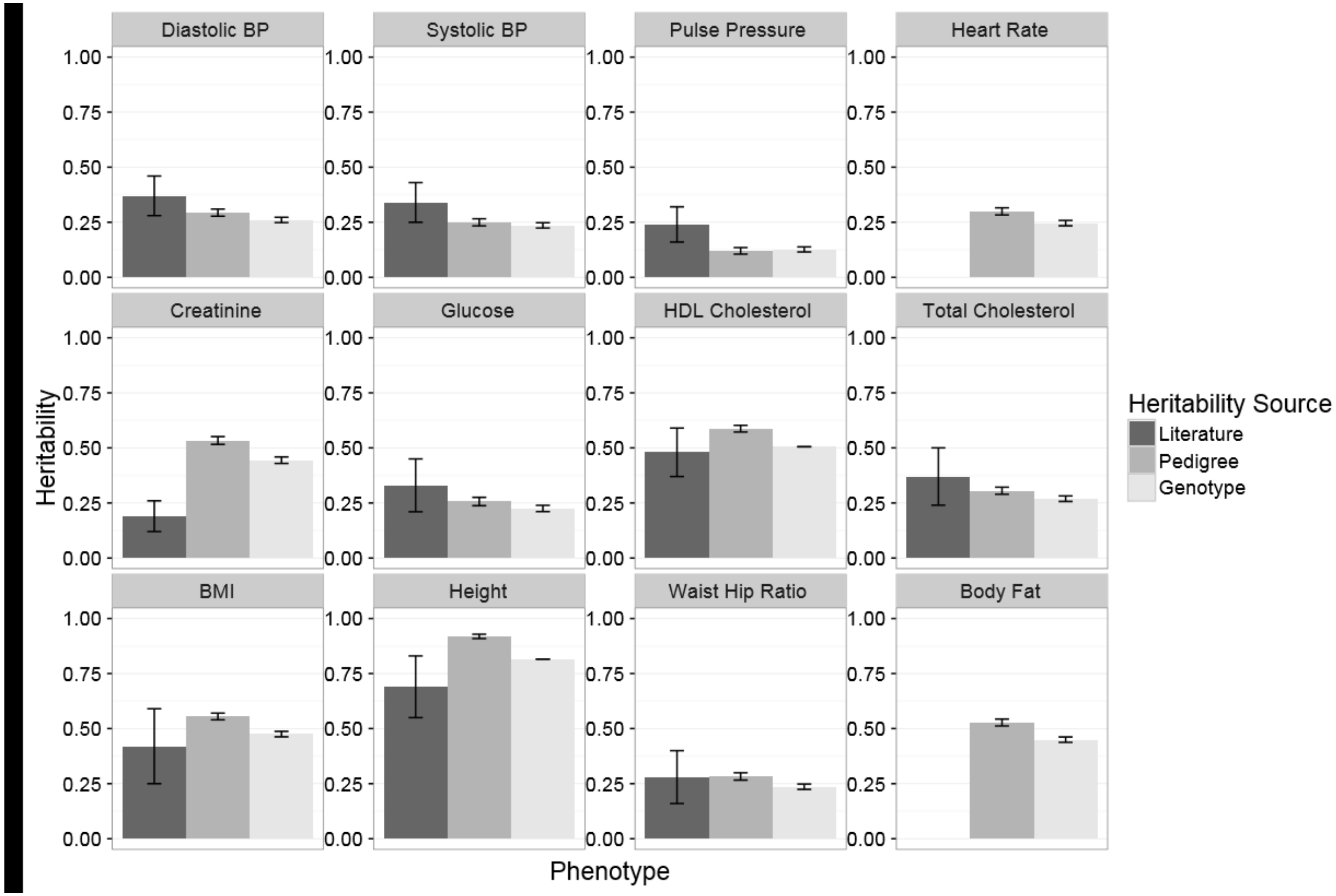

Additional file 2: Figure S1 - Phenotype Heritabilities

Heritabilities were estimated using the social pedigrees (no genetic data was used here) as well as using genotype data. These are compared to the heritability estimates quoted in the literature (references in Additional file Table 1). Error bars show the standard error of the mean.

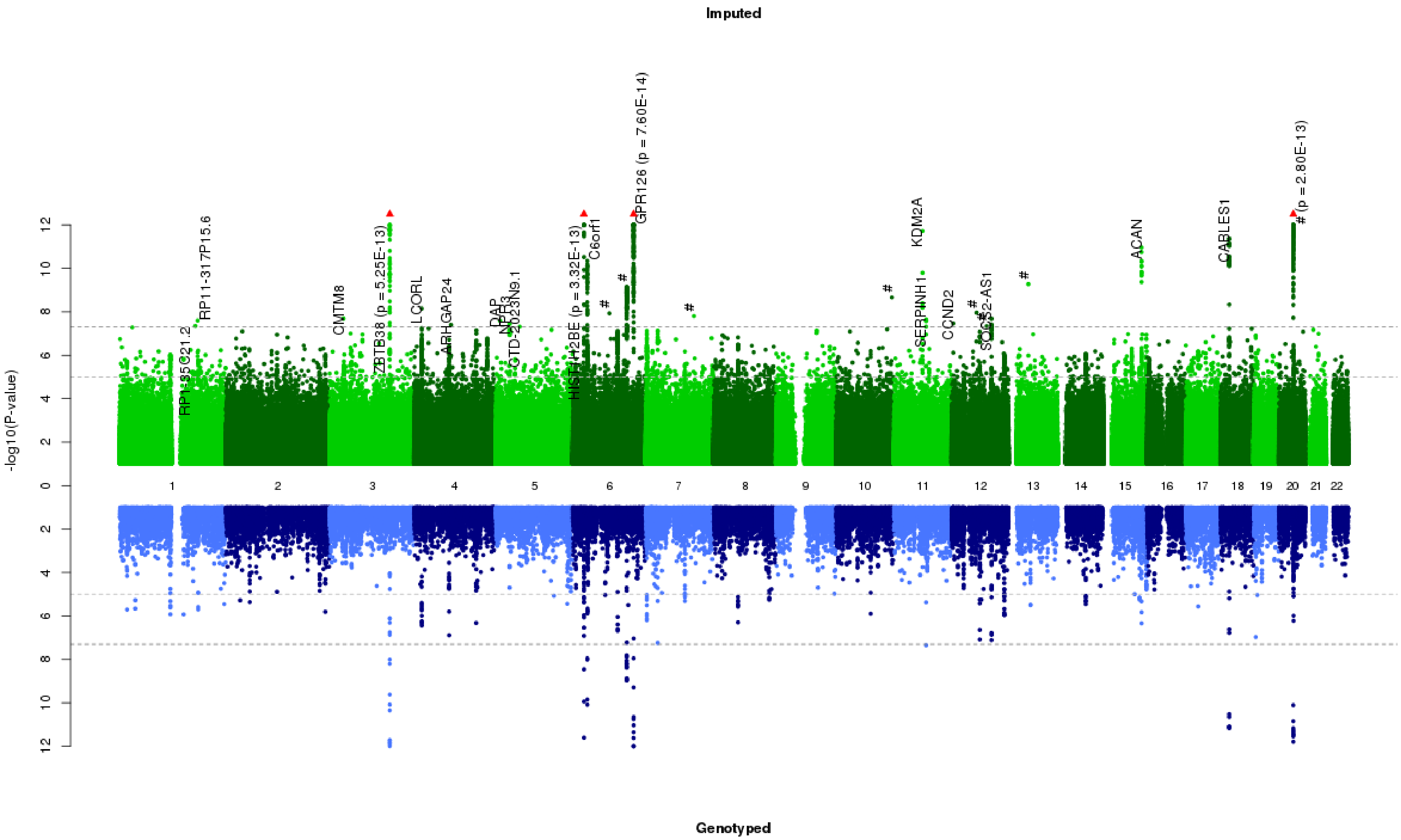

Additional file 2: Figure S2 - Miami plot for height. The # symbol denotes a hit in an intergenic region.

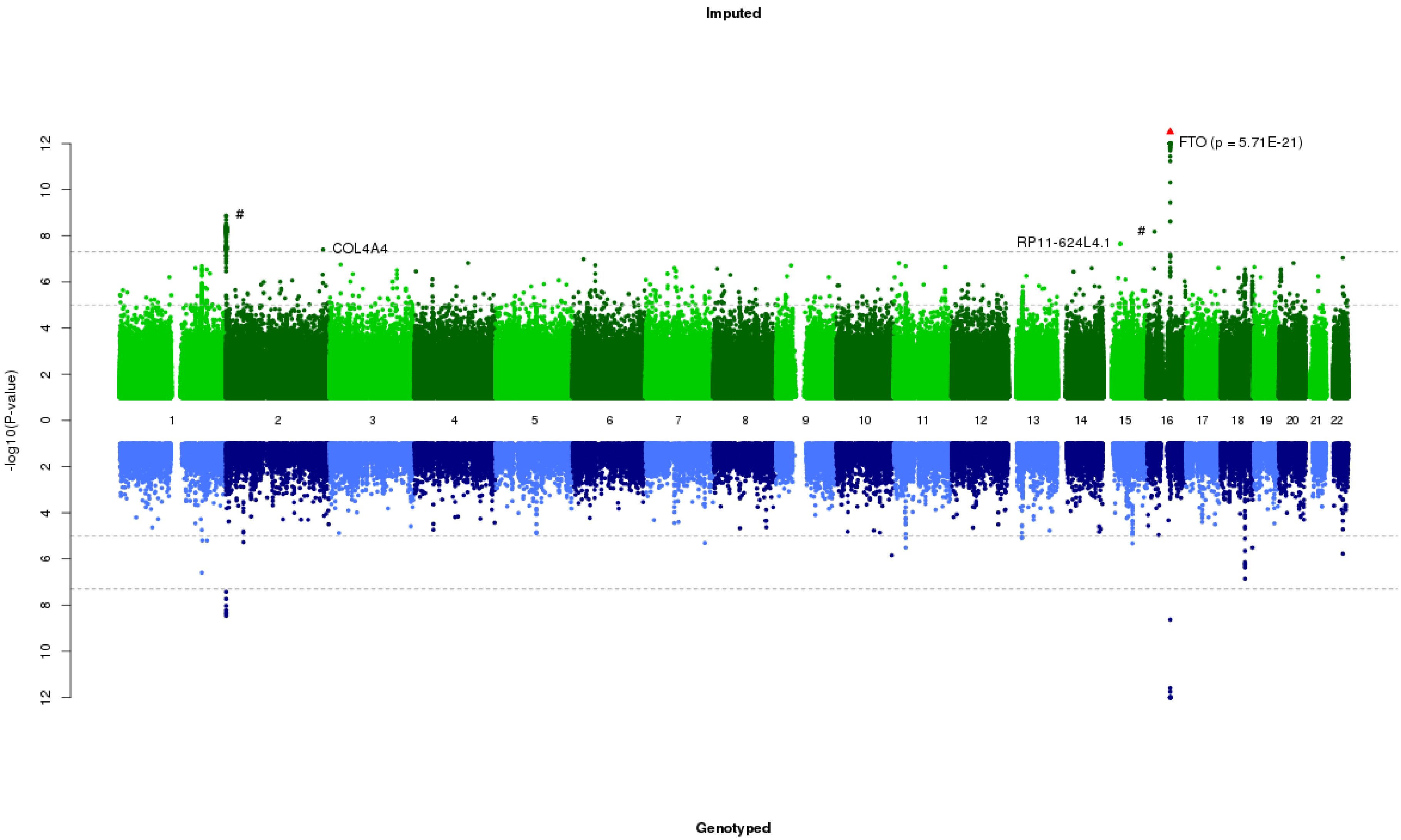

Figure

Additional file 2: Figure S3- Miami plot for BMI. The # symbol denotes a hit in an intergenic region.

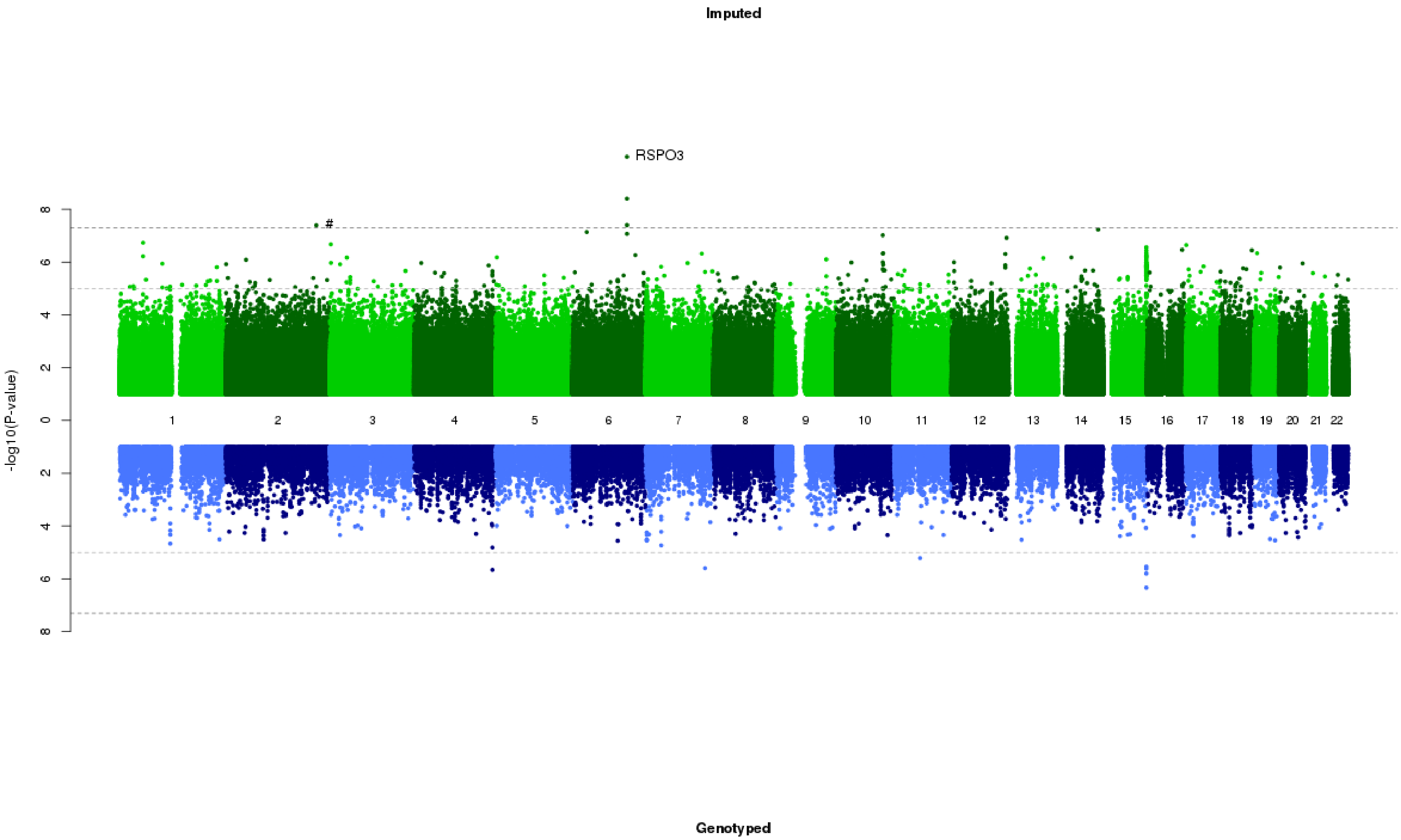

Additional file 2: Figure S4 - Miami plot for waist circumference. The # symbol denotes a hit in an intergenic region

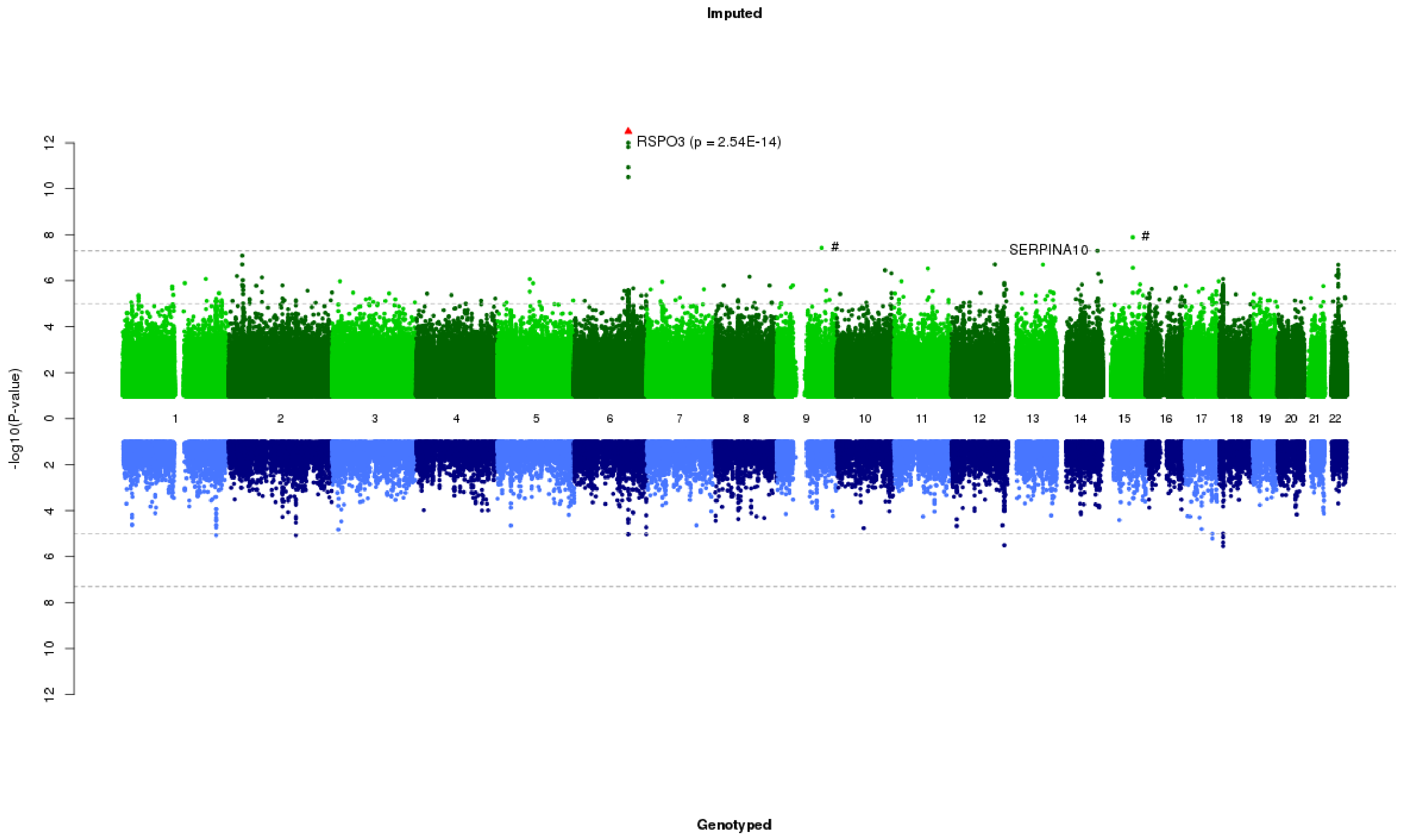

Additional file 2: Figure S5- Miami plot for waist-to-hip ratio. The # symbol denotes a hit in an intergenic region.

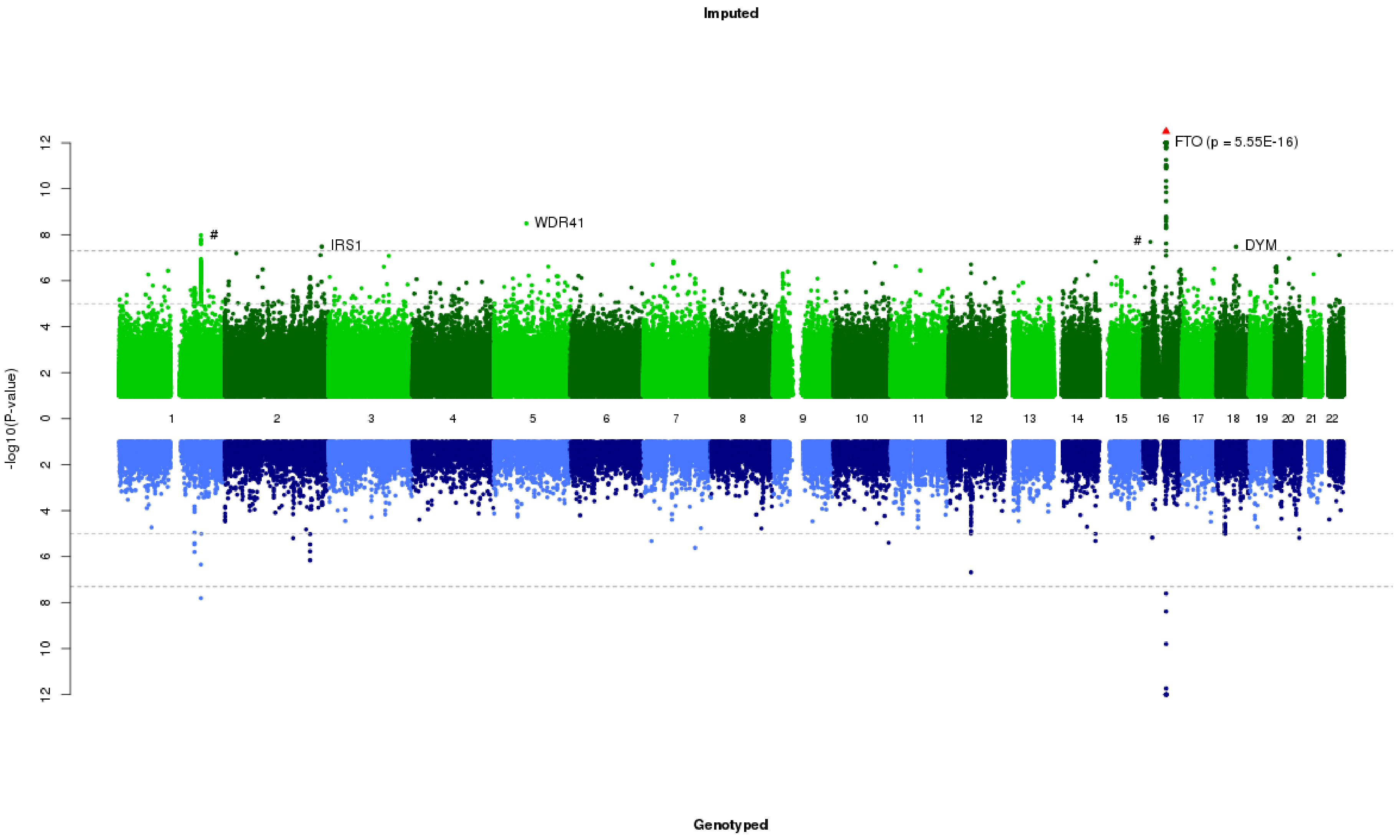

Additional file 2: Figure S6 - Miami plot for body fat percentage. The # symbol denotes a hit in an intergenic region.

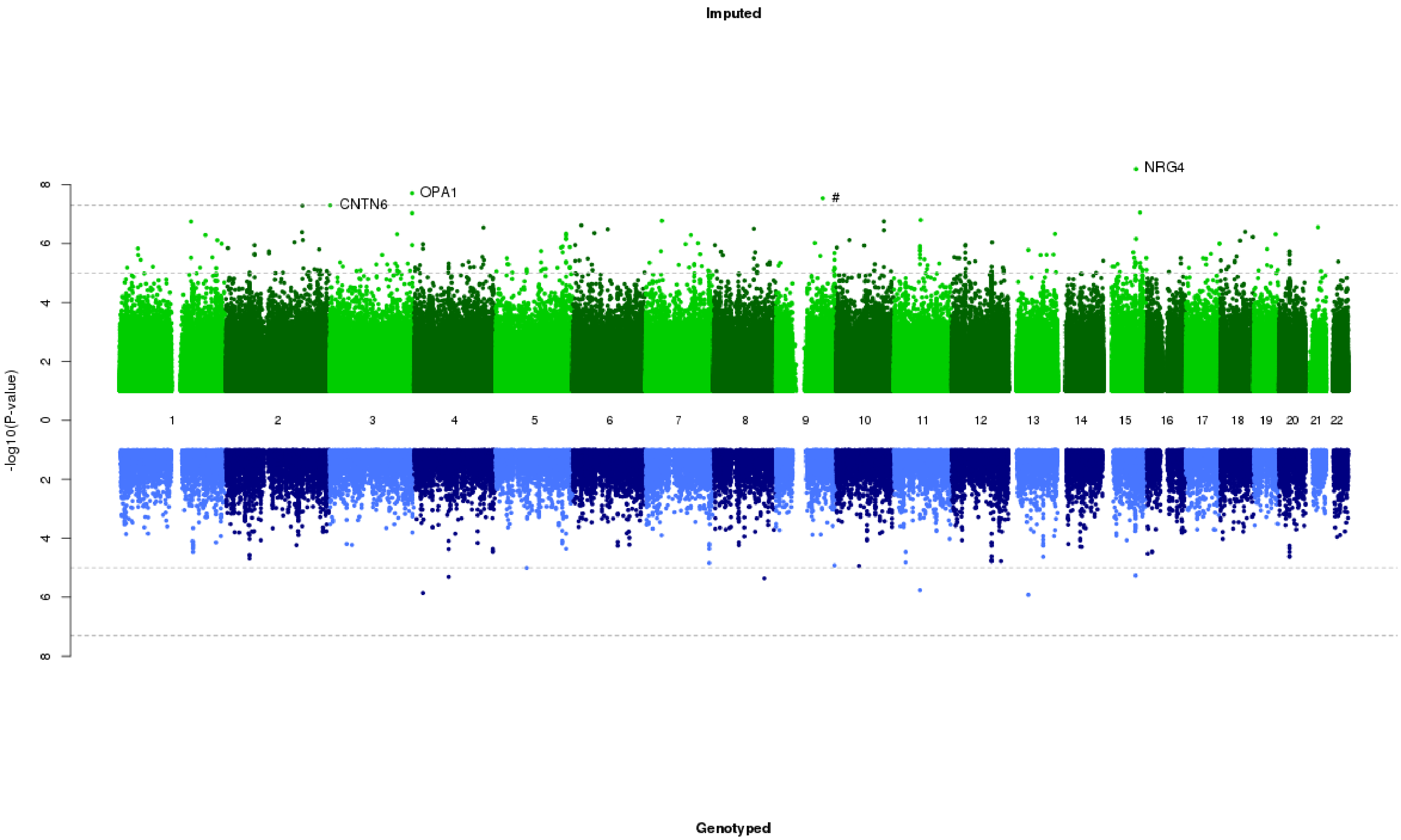

Additional file 2: Figure S7 -Miami plot for diastolic blood pressure. The # symbol denotes a hit in an intergenic region.

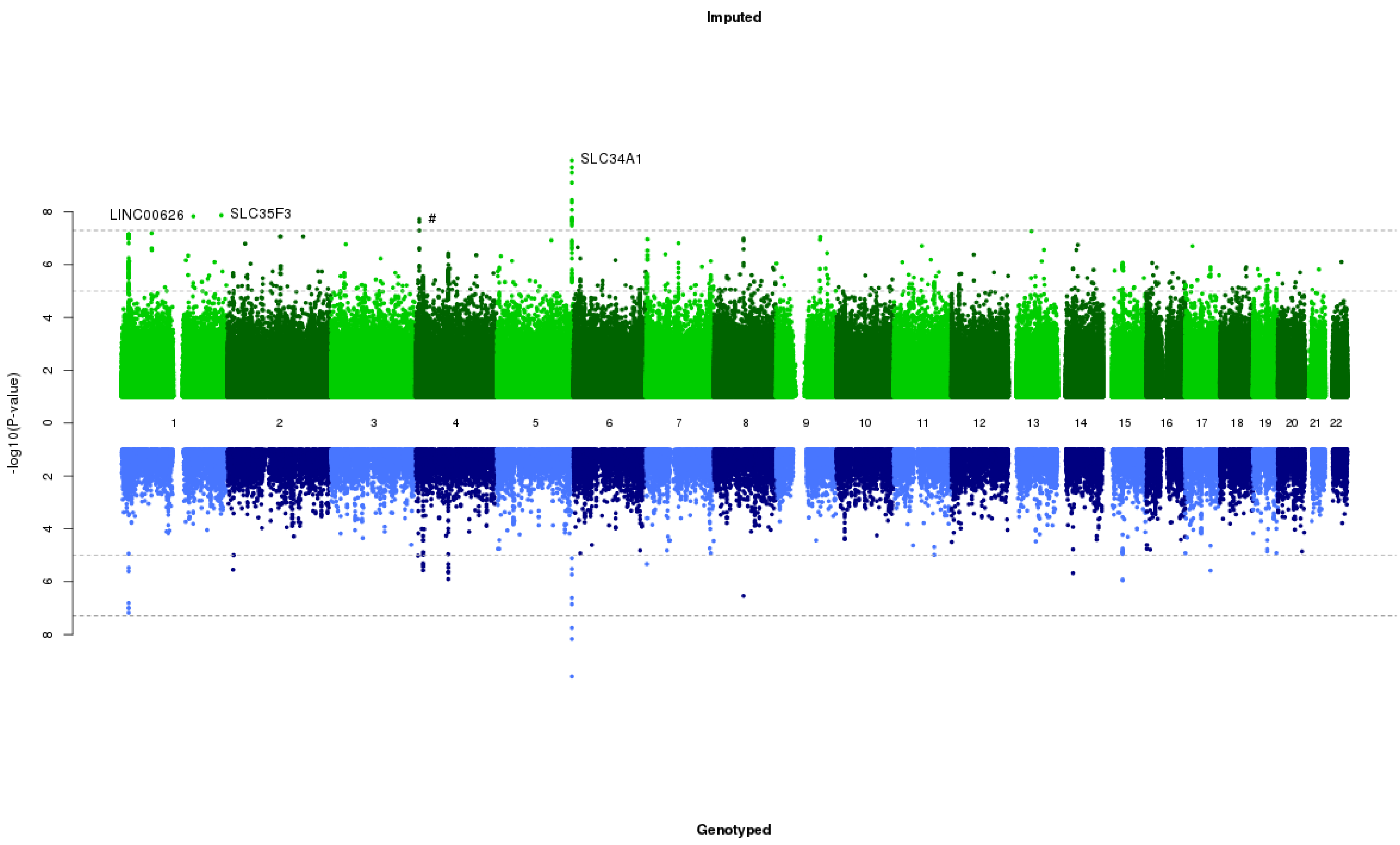

Additional file 2: Figure S8 - Miami plot for creatinine. The # symbol denotes a hit in an intergenic region.

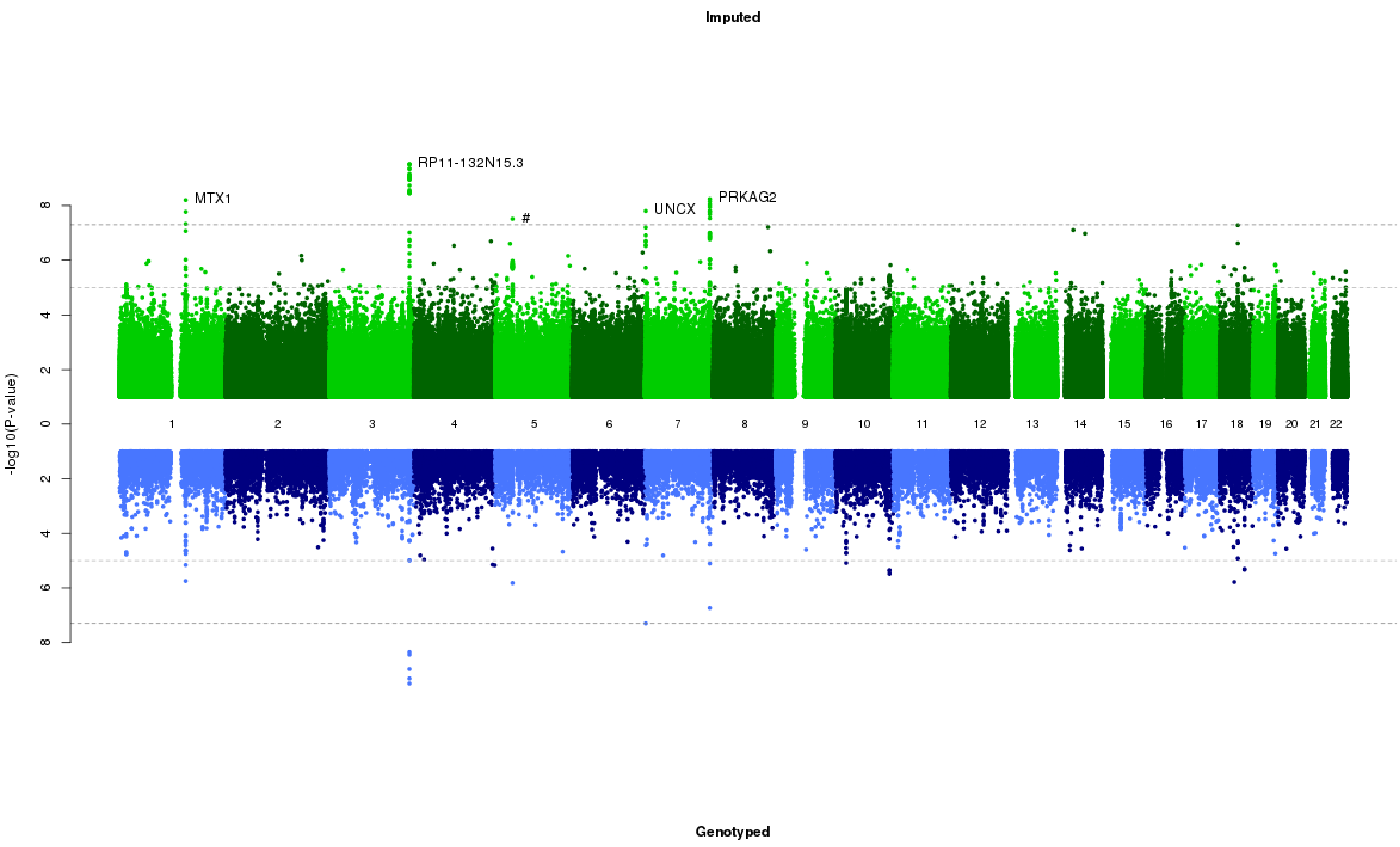

Additional file 2: Figure S9 - Miami plot for urea. The # symbol denotes a hit in an intergenic region.

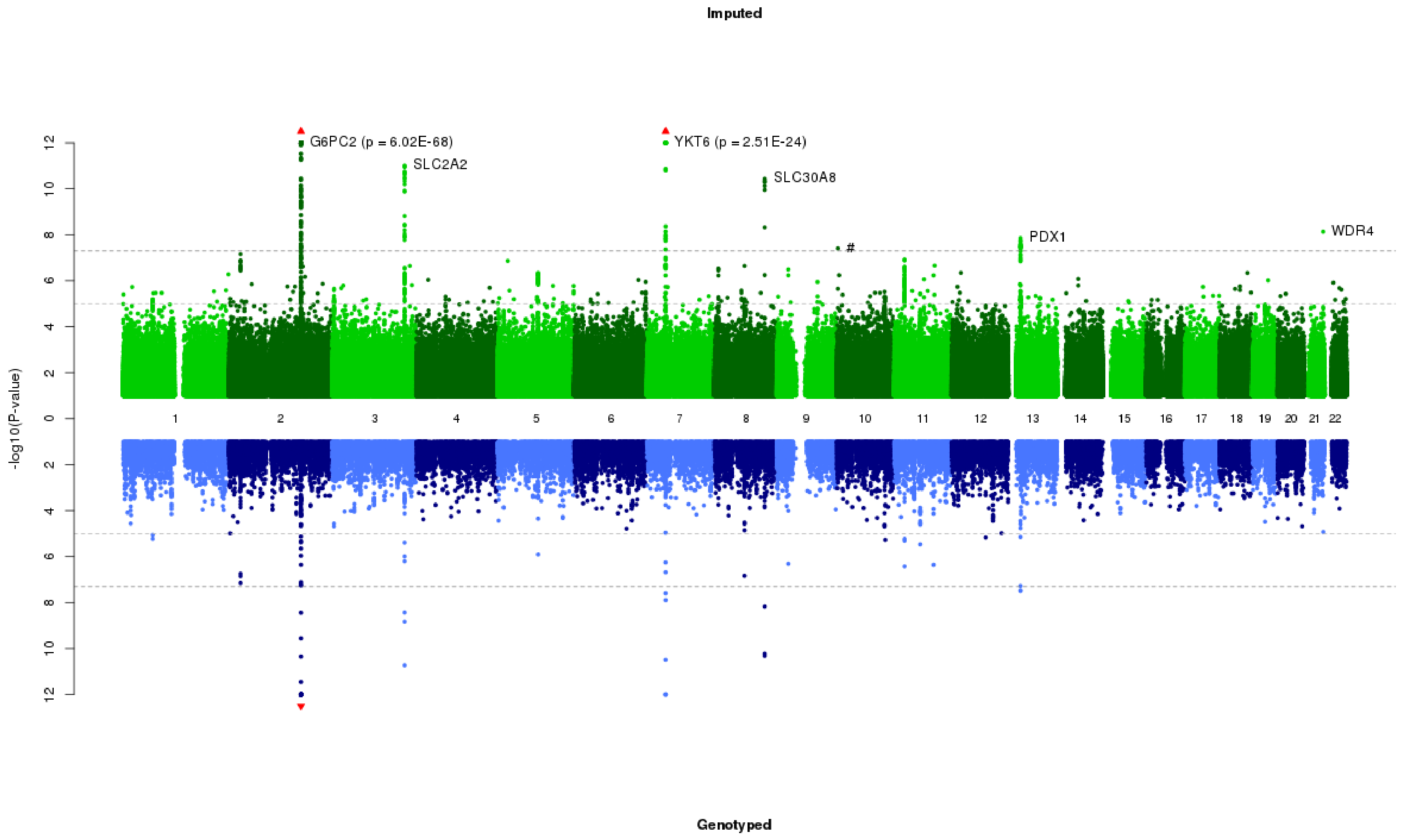

Additional file 2: Figure S10 - Miami plot for fasting glucose (all individuals included). The y-axis has been truncated to show only SNPs with p-values less than 10^{-12}. Red arrows denote loci with additional markers with lower p-values. The # symbol denotes a hit in an intergenic region.

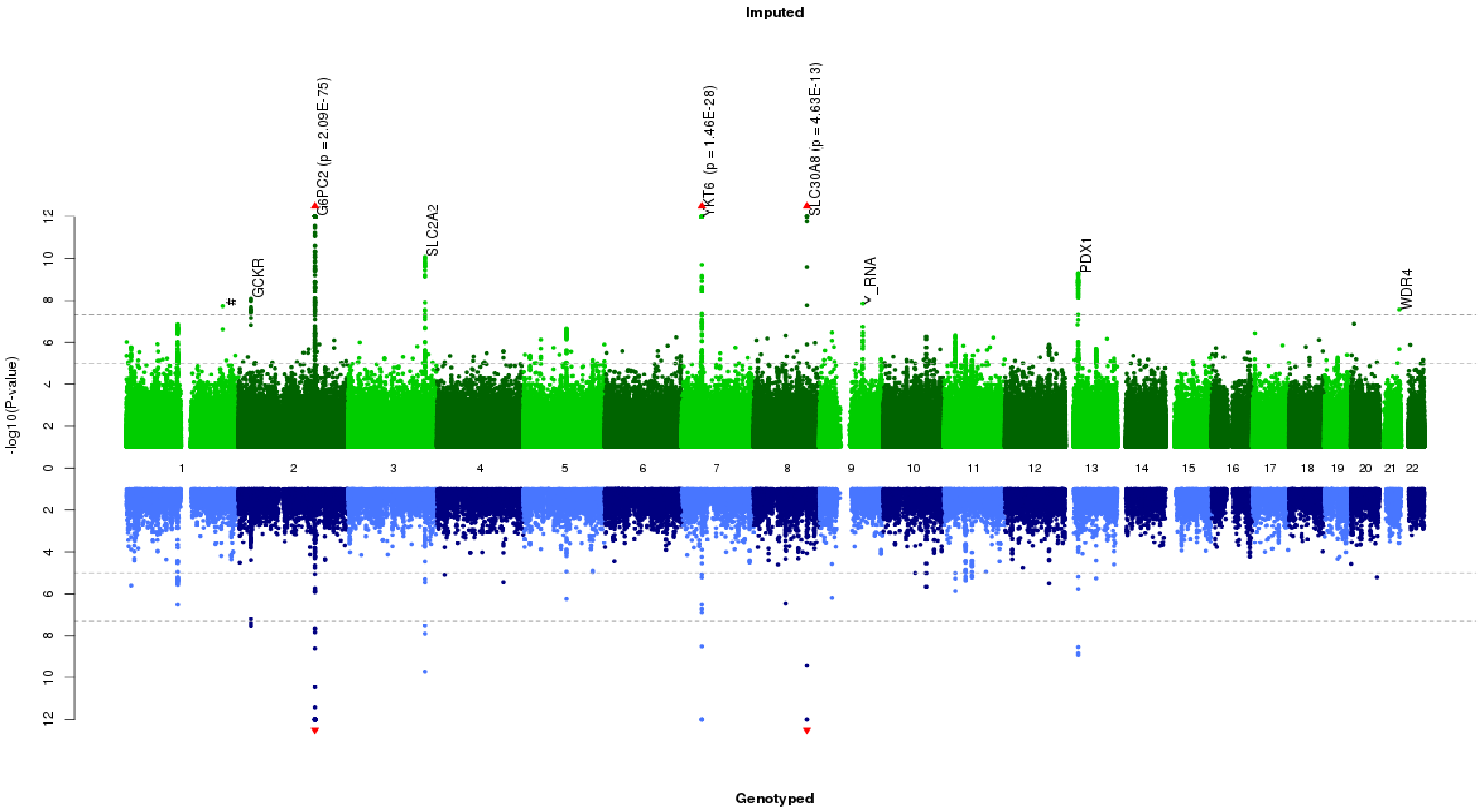

Additional file 2: Figure S11 - Miami plot for fasting glucose (excluding diabetics and measurements of >7mmol/L). The y-axis has been truncated to show only SNPs with p-values less than 10^{-12}. Red arrows denote loci with additional markers with lower p-values. The # symbol denotes a hit in an intergenic region.

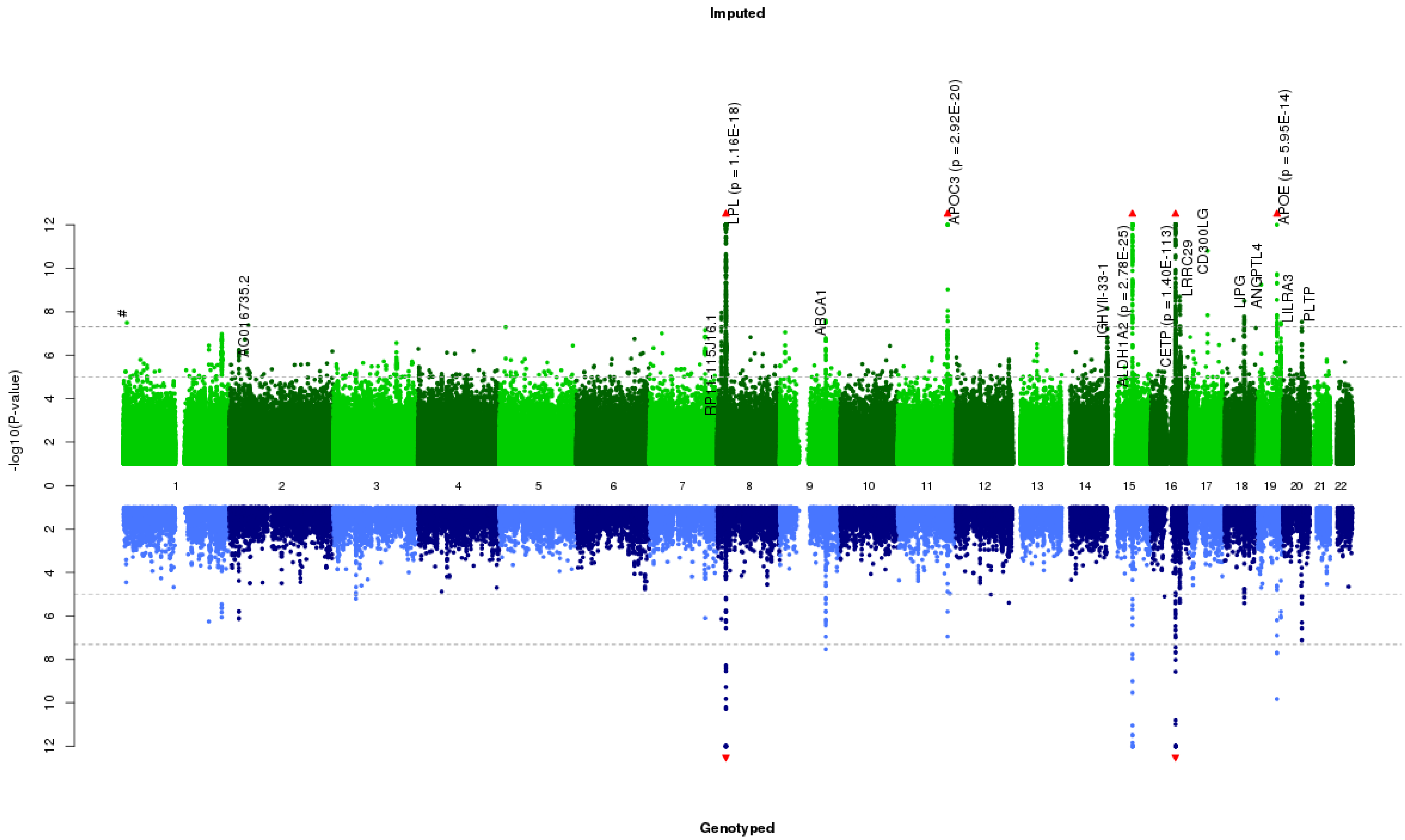

Additional file 2: Figure S12 - Miami plot for HDL cholesterol. The y-axis has been truncated to show only SNPs with p-values less than 10^{-12}. Red arrows denote loci with additional markers with lower p-values. The # symbol denotes a hit in an intergenic region.

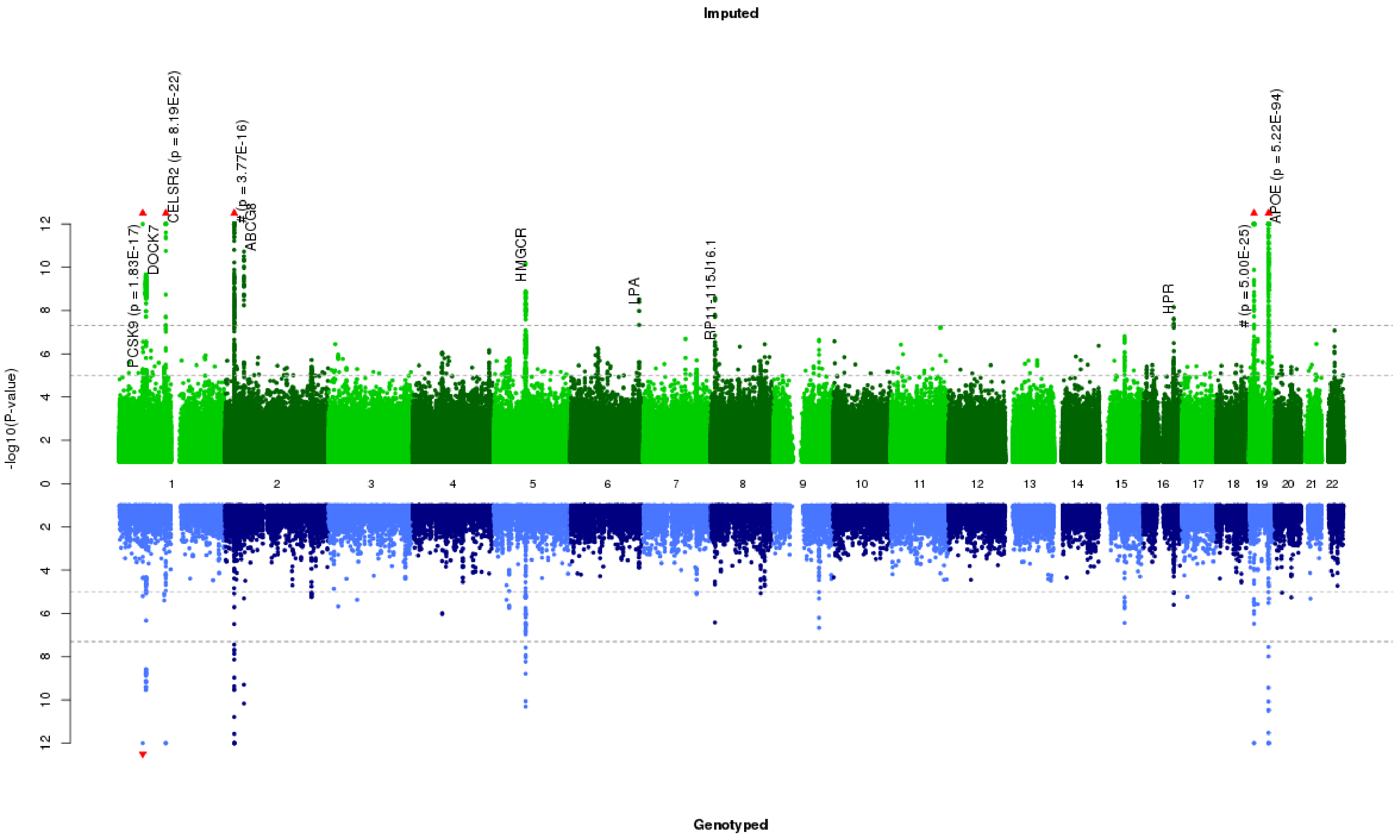

Additional file 2: Figure S13 - Miami plot for total cholesterol. The y-axis has been truncated to show only SNPs with p-values less than 10^{-12}. Red arrows denote loci with additional markers with lower p-values. The # symbol denotes a hit in an intergenic region.

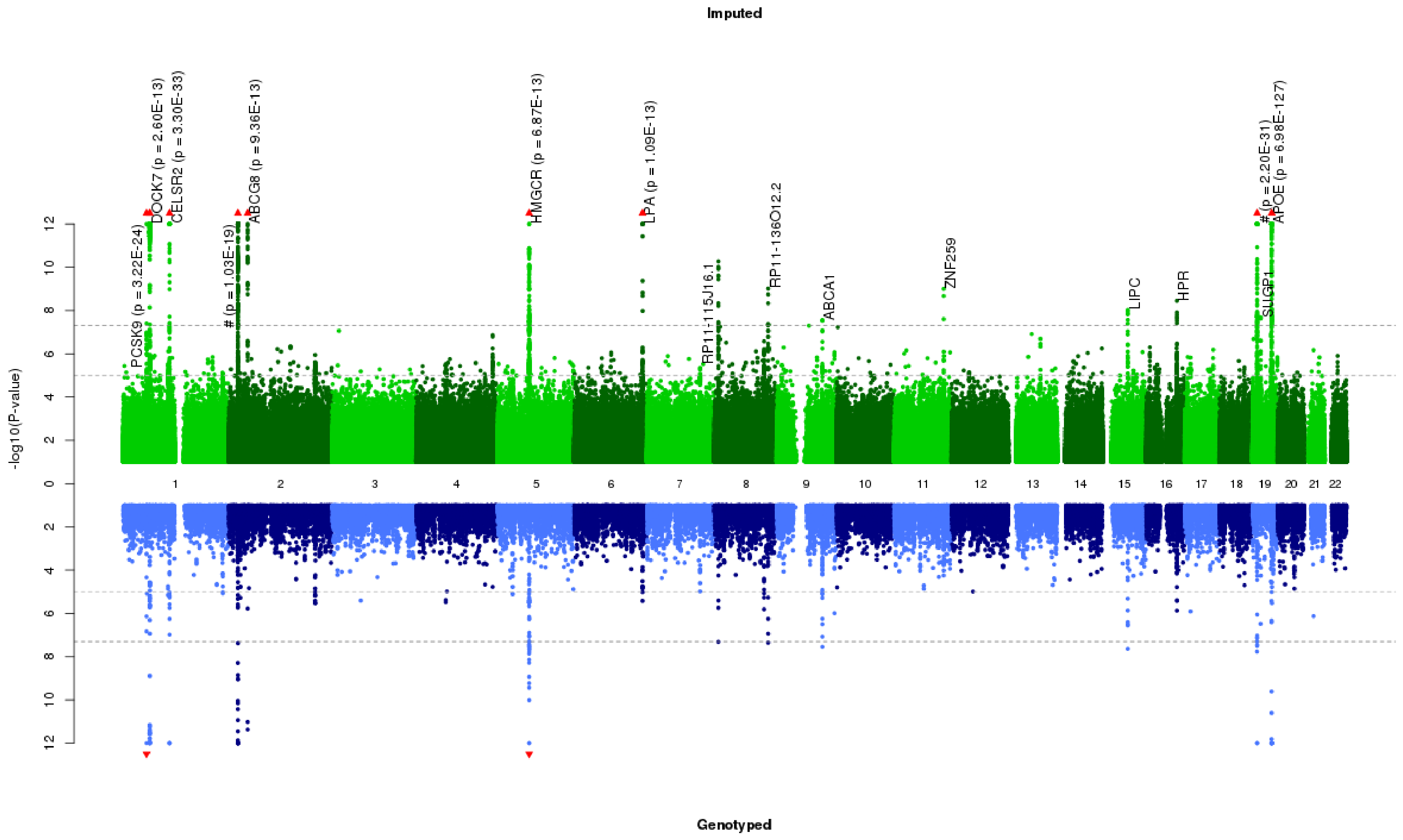

Additional file 2: Figure S14- Miami plot for total cholesterol adjusted for statin use. The y-axis has been truncated to show only SNPs with p-values less than 10^{-12}. Red arrows denote loci with additional markers with lower p-values. The # symbol denotes a hit in an intergenic region.

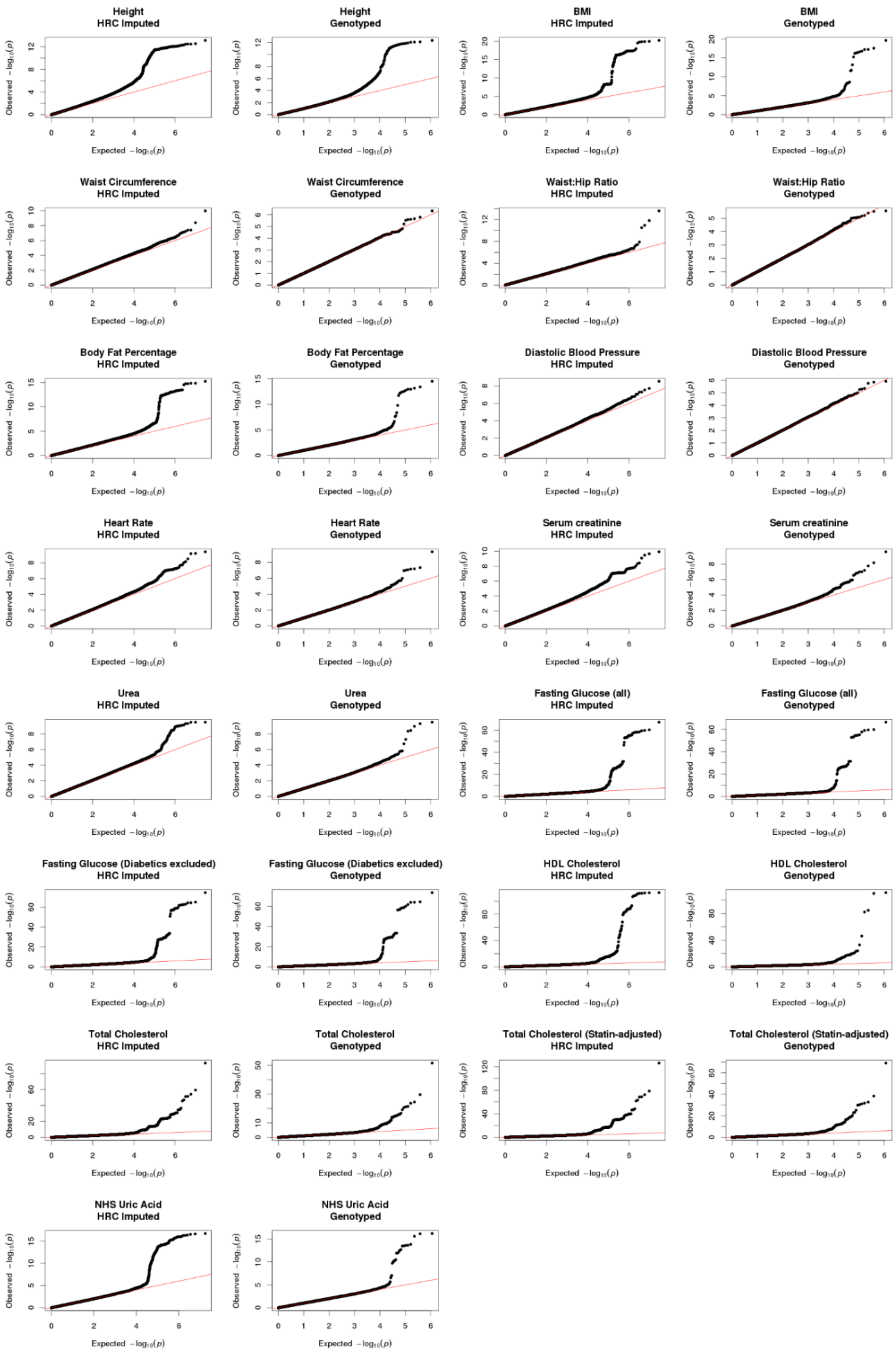

Additional file 2: Figure S15 - QQ plots for all traits analysed

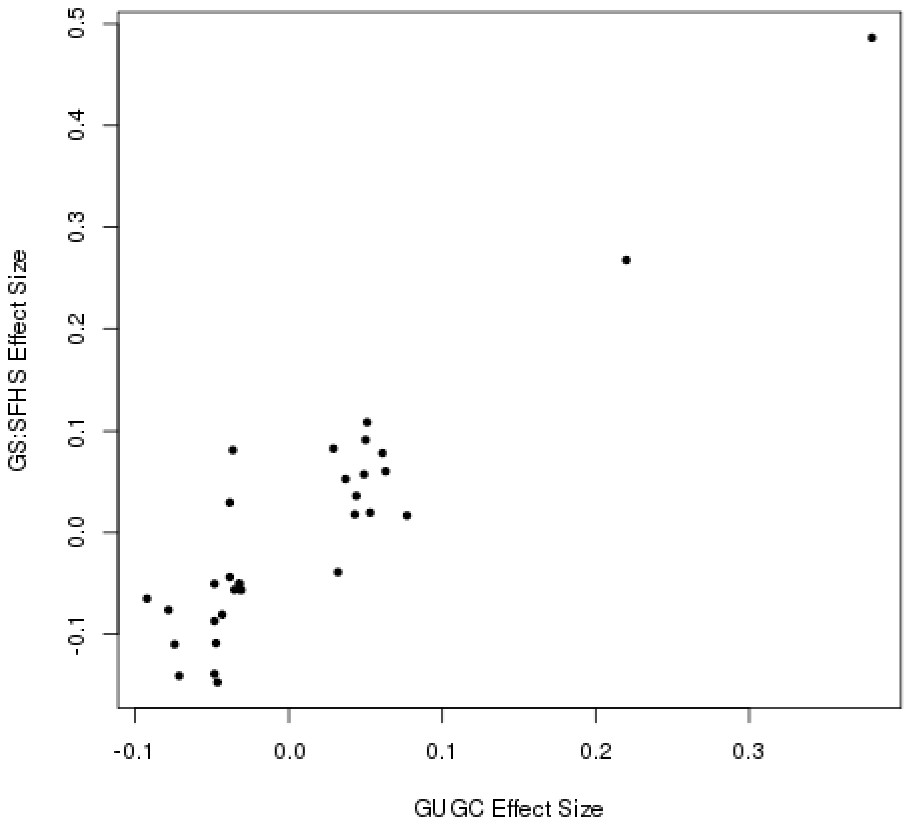

Additional file 2: Figure S16 - Comparison of effect sizes for GUGC top hits in the GS:SFHS EHR analysis
